# Tracking the spatiotemporal variations of statistically independent components involving enrichment of rare-earth elements in deep-sea sediments

**DOI:** 10.1038/srep29603

**Published:** 2016-07-22

**Authors:** Kazutaka Yasukawa, Kentaro Nakamura, Koichiro Fujinaga, Hikaru Iwamori, Yasuhiro Kato

**Affiliations:** 1 Department of Systems Innovation, School of Engineering, The University of Tokyo, 7-3-1 Hongo, Bunkyo-ku, Tokyo 113-8656, Japan; 2 Ocean Resources Research Center for Next Generation, Chiba Institute of Technology, 2-17-1 Tsudanuma, Narashino, Chiba 275-0016, Japan; 3 Department of Solid Earth Geochemistry, Japan Agency for Marine-Earth Science and Technology (JAMSTEC), 2-15 Natsushima-cho, Yokosuka, Kanagawa 237-0061, Japan; 4 Department of Earth and Planetary Sciences, School of Science, Tokyo Institute of Technology, 2-12-1 Oo-Okayama, Meguro-ku, Tokyo 152-8550, Japan; 5 Frontier Research Center for Energy and Resources, School of Engineering, The University of Tokyo, 7-3-1 Hongo, Bunkyo-ku, Tokyo 113-8656, Japan; 6 Research and Development Center for Submarine Resources, Japan Agency for Marine-Earth Science and Technology (JAMSTEC), 2-15 Natsushima-cho, Yokosuka, Kanagawa 237-0061, Japan

## Abstract

Deep-sea sediments have attracted much attention as a promising resource for rare-earth elements and yttrium (REY). In this study, we show statistically independent components characterising REY-enrichment in the abyssal ocean that are decoded by Independent Component Analysis of a multi-elemental dataset of 3,968 bulk sediment samples from 101 sites in the Pacific and Indian oceans. This study for the first time reconstructs the spatiotemporal variations of the geochemical signatures, including hydrothermal, hydrogenous, and biogenic calcium phosphate components that were closely involved in the formation of REY-rich mud over the past 65 million years. An underlying key factor of significant REY-enrichment is a sufficiently low sedimentation rate that enables the mud to accumulate REY from seawater. In the early Cenozoic, a remarkably small supply of aeolian dust, compared with any other time and region, facilitated the deposition of very high-grade REY-rich mud in the South Pacific. This indicates an important link between the genesis of the seafloor mineral resources and Earth’s dynamic phenomena such as climate change and plate tectonics.

Rare-earth elements and yttrium (REY) play essential roles in a variety of highly advanced devices and green technologies. Therefore, a very rapid increase in future global demand for REY is expected^1^. Previous reports have demonstrated the widespread distribution of REY-rich mud, or deep-sea sediments containing high concentrations of REY, in the Pacific^2^ and Indian oceans^3^,^4^. Because these sediments have potential economic value as a favourable source of REY, understanding the global distribution of REY-rich mud has become an important issue.

Characterising the REY-enrichment in deep-sea sediments is key in determining areas of high resource potential worthy of detailed exploration and for revealing latent relationships between Earth’s dynamic systems and marine mineral resources. The geochemical signatures of factors closely related to the genesis of REY-rich mud are likely preserved as characteristic bulk chemical compositions of the sediments. In order to decode the commingled signals in various types of deep-sea sediments that originated from multiple source materials and physicochemical processes, multivariate statistical analyses that can treat multi-elemental information have long been employed^5^,^6^,^7^,^8^.

Although application of traditional multivariate statistical analyses such as Principal Component Analysis (PCA) or Factor Analyses (FA) to geochemical data can be insightful, these methods have limitations for use and cannot be applied to certain datasets such as those discussed here. Both PCA and FA transform the data using only the mean and variance, or first- and second-order statistics. This implies that the extracted new variables, known as principal components or common factors, are mutually independent in the true sense only when the observed data constitute a multivariate Gaussian distribution^9^. In fact, the sediment data of the Pacific and Indian oceans exhibit large skewness and multimodal distributions (Supplementary Fig. S1); therefore, application of PCA or FA to extract independent features is not necessarily appropriate^10^,^11^.

The aforementioned constraints notwithstanding, a number of previous works have demonstrated fruitful results by applying multivariate analyses to datasets of tens to hundreds of samples. Here, we build upon these studies of marine sediments and expand our perspective to examine global-scale features by using a new statistical approach on a massive geochemical dataset. We construct a hemisphere-scale compositional dataset of 3,968 bulk sediment samples from 82 sites in the Pacific Ocean and 19 sites in the Indian Ocean (Fig. 1). Moreover, we employ Independent Component Analysis (ICA) to identify the geochemical signatures hidden in the huge dataset of deep-sea sediments. ICA is a relatively new computational statistical technique established in the fields of neuroscience and information science during the past quarter century^9^; its utility has also been recognised in the geochemical field^2^,^11^,^12^,^13^,^14^,^15^. ICA can extract original independent source signals from observed signals on the basis of a fundamental assumption that the observed data consist of mutually independent source signals showing non-Gaussian distributions^9^. As the result of ICA, the original multi-elemental data can be expressed by a combination of independent components (ICs) corresponding to geochemical materials or processes statistically independent of each other.

## Results

### Data structure of bulk sediment geochemistry

Scatter plots of the major element oxide and REY contents of all samples are shown in Fig. 2 and in Supplementary Fig. S2. Most of the plots exhibit data distributions that are sharply cut off at the edges, often with minor elongated structures, which are far from the theoretical distribution of data that constitute a multivariate Gaussian distribution^9^. Indeed, histograms of the elemental contents indicate multimodal, concave, or long-tailed distributions (Supplementary Fig. S1), reflecting the inherent non-Gaussian structures. These observations justify the application of ICA for evaluating the origin of the compositional variations in various deep-sea sediment samples.

### Independent Component Analysis

ICA was performed by using 11 variables, including the contents of SiO_2_, TiO_2_, Al_2_O_3_, Fe_2_O_3_, MnO, MgO, CaO, K_2_O, P_2_O_5_, total REY except Ce (ΣREY), and Ce. Cerium is handled separately because its geochemical behaviour differs from that of other REY elements owing to its specific redox sensitivity^16^,^17^ (Supplementary Fig. S3). The ICA result (Fig. 2, Supplementary Figs S4–S6) indicates that the 11-dimensional chemical compositions of bulk sediment samples can be successfully expressed by 7 ICs that collectively account for 97.9% of the total sample variance. It should be noted that the ICs are expressed as a set of new base vectors in the compositional spaces along which the chemical compositions of the samples shift independently rather than as geometric points with specific compositions, often referred to as end-members^12^,^14^. The ICs cannot be ranked simply according to their proportion of data variance as in the case of PCA because the ICs are independent. Thus, the numbering of ICs is commutative, and there is no way to measure the relative importance between ICs^12^. The effects of the uncertainties in the chemical analysis on the ICA results were assessed by random data perturbation analysis^11^,^14^ (Supplementary Fig. S7). With the exception of IC2 showing sensitive behaviour to the data uncertainty, it was confirmed that the remaining six ICs converged well, and were thus considered to be robust signals.

### Characterization of geochemical independent components

Interpretation of the result of our geochemical ICA was conducted on the basis of IC scores and IC loadings (Supplementary Figs S4–S6; Supplementary Tables S1 and S2). IC scores correspond to the coordinate values of the sample data in the IC space, whereas IC loadings represent the relative contributions of each element to the individual ICs. By linear transformation, each IC can be projected as a vector in the compositional space (Fig. 2a,b; Supplementary Fig. S2), or the compositional data of each sample can be plotted in the space spanned by the ICs^11^,^12^,^13^,^14^ (Fig. 2c–e; Supplementary Fig. S4). More detailed explanations of IC scores and loadings are given in the Supplementary Information.

IC3 had positive loading only in CaO and negative loadings in all other elements (Supplementary Fig. S6), which is reflected in the compositional change to Ca-rich samples from the others along IC3 (Fig. 2a). In the IC space, the IC3 axis clearly discriminated the Ca-rich samples from the others (Fig. 2c; Supplementary Fig. S4c,d). These features indicate that IC3 extracts a mixing trend between biogenic calcium carbonate and other components. In the same manner, IC5 had clearly positive loading only in SiO_2_ (Supplementary Fig. S6), and Si-rich samples were distributed along the IC5 vector (Fig. 2a,c). In addition, the projected data of chert^18^ as a reference material plotted in the same domain as samples having highly positive IC5 scores in the IC space (Fig. 2c; Supplementary Fig. S4c). Thus, IC5 corresponded to a mixing trend between biogenic silica and others. IC6 had positive loadings in TiO_2_, Fe_2_O_3_, and MgO (Supplementary Fig. S6). Because Mg- and Ti-rich samples and reference volcanic rocks were simultaneously projected in the positive domain of IC6 (Fig. 2a,c; Supplementary Fig. S4d), this IC is considered to represent the influence of the volcaniclastic component. Terrigenous detrital aluminosilicates (e.g. Chinese loess or Australian dust supplied as aeolian dust) did not show up in the ICA result explicitly, despite their prevalence in deep-sea sediments. This is because the compositional variation in terrigenous detrital materials is not as large as that in the other components. In contrast to the above three ICs, a clear diagnostic feature of IC2 was not recognised in the IC loadings or in the data distribution in the IC space. Considering its lower robustness to data uncertainty, IC2 might reflect a noisy structure inherent in the present dataset or some unknown factor barely characterised by major elements and REY. More comprehensive and higher dimensional analysis using various trace elements might help to identify the geochemical implication of this IC.

The remaining three ICs (i.e. IC1, IC4, and IC7) had positive loadings of REY (Supplementary Fig. S6), and their slopes showed clear REY-enrichment in the elemental compositional spaces (Fig. 2b). Nevertheless, in the three-dimensional IC space spanned by these ICs (Fig. 2d), the projected data distribution exhibited distinct orthogonality. These features indicate that the REY-controlling factor in deep-sea sediment can be resolved into three statistically ICs and that each REY-rich mud sample was generated by a combination of the three components in different degrees.

IC7 had positive loadings of MnO, Fe_2_O_3_, P_2_O_5_, and ΣREY in decreasing order (Supplementary Fig. S6), and the reference data of hydrothermal Fe- and Mn-rich sediments were always projected in the same domain as high-IC7 samples in IC spaces (Fig. 2d; Supplementary Fig. S4b). Thus, IC7 corresponds to the hydrothermal component that directly precipitates from a hydrothermal plume and results in significant Fe- and Mn-enrichment in the sediment^19^. IC7 is comparable to the Fe-rich trend associated with the enrichment of REY in the Fe_2_O_3_–ΣREY diagram^2^. A probable origin of the Fe-, Mn-, P- and REY-enriched sediments showing high IC7 scores was suggested to be a scavenging mechanism by Fe-oxyhydroxides in hydrothermal plumes which takes up various elements, including P and REY, from ambient seawater during advection through the water column and after settling on the seafloor^19^,^20^.

Both IC1 and IC4 had generally loading patterns similar to highly positive P_2_O_5_ and ΣREY, although only IC1 showed prominently positive Ce loading together with positive Mn loading (Supplementary Fig. S6). In the two-dimensional IC space spanned by IC1 and IC4 (Fig. 2e), the samples showing very high REY concentrations (ΣREY + Ce > 1,000 ppm), generally zeolitic clay or pelagic red clay in lithology^2^,^3^,^4^, were distributed in the positive domains of each IC. For IC1, the reference data of both biogenic calcium phosphate (Ca-phosphate) and Fe–Mn oxides such as ferromanganese crusts and nodules were projected in the same domain as the high-IC1 REY-rich mud samples (Fig. 2e). A number of previous studies have demonstrated that biogenic Ca-phosphate constituting the bones and teeth of marine vertebrates shows very high concentrations of REY and regulates the bulk REY abundance in pelagic sediments^21^,^22^,^23^,^24^,^25^,^26^. Moreover, hydrogenetically precipitated Mn oxides (δ–MnO_2_) uptake Ce in seawater preferentially via oxidation of Ce (III) to Ce (IV)^27^,^28^,^29^, which can explain the very high loading of Ce exhibited by IC1. Hence, IC1 represents REY-rich mud concentrating both REY-enriched biogenic Ca-phosphate and Ce-enriched Fe–Mn oxides. For IC4, however, the biogenic Ca-phosphate was projected in the positive domain of IC4, although the Fe–Mn oxides were on the opposite side (IC4 < 0) in the IC space (Fig. 2e). Therefore, IC4 represents REY-rich mud with an abundance of biogenic Ca-phosphate but with a deficiency of hydrogenous Fe–Mn oxides.

### Checking the statistical robustness of independent components

In addition to the analytical error, several factors that potentially affect the ICA results were further checked individually, such as outliers in the dataset, the variability of the sample subsets between the Pacific and Indian oceans, and the recalculation of raw data to sum to 100%. The results of the sensitivity tests for such factors indicated that the geochemical ICs described above were statistically robust and valid (Supplementary Figs S8–S16).

To examine the manner in which the REY-enrichment ICs can be affected by dilution effects and by the element menu analysed, we performed additional ICA on samples without significant dilutions from biogenic carbonate and silica by using the selective variables of Fe_2_O_3_, MnO, CaO, P_2_O_5_, and all of the separated REY elements. This additional ICA (*n* = 1,575) resulted in extraction of essentially the same ICs as the original REY-enrichment ICs, i.e. IC1, IC4, and IC7 (Supplementary Fig. S17).

The results of these robustness checks show that the original ICA result sufficiently extracted the essential geochemical features of both the REY-enrichment ICs and others from the comprehensive dataset.

### Spatiotemporal distributions of independent components

By using the sedimentary age inferred mainly from available biostratigraphic information (see Supplementary Information) and from palaeogeographic reconstruction^30^,^31^, we visualised the spatiotemporal distributions of IC scores over the past 65 million years (Myr; Fig. 3). It should be noted that we excluded samples in which the age could not be estimated because of a hiatus, coring disturbance, or paucity of key fossils for age determination. In addition, the IC score distributions do not directly correspond to the present-day seafloor-surface sediment because the IC scores of old deeply buried sediments are merely projected onto the modern geography. However, the projected geographical patterns of IC scores provided additional information on the characteristics of each IC.

The signals of IC3 were strongest around mid-ocean ridges and oceanic plateaus (Fig. 3b), corresponding to a significant deposition of biogenic calcium carbonate above the carbonate compensation depth (CCD). IC5 had the highest values in the equatorial Pacific Ocean, showing the highest surface productivity in the present ocean (Fig. 3d). However, the highest IC5 score was derived from the sediment at Deep Sea Drilling Project (DSDP) Site 163 deposited 30 to 50 million years ago (Ma). In addition, relatively high IC5 intensities at DSDP Sites 166 and 65 originated from sediment deposited 5–25 Ma and 0–15 Ma, respectively. These results suggest that the high primary productivity in the eastern to central equatorial Pacific has been maintained throughout the Cenozoic. The high-IC6 samples correspond to sediments of 35–40 Ma at DSDP Site 45 in the central North Pacific and those of ~65 Ma at DSDP Site 219 off southwestern India (Fig. 3e). These prominent IC6 signals can be associated with two major volcanic edifices: the Hawaiian hotspot and the Deccan Traps^32^.

IC1 exhibited remarkably high scores of 8–15 at DSDP Site 596 in the central South Pacific before ~25 Ma and moderately high scores of 1–3 in the central and eastern North Pacific throughout the Cenozoic era (Fig. 3a). The latter broadly overlaps the distribution of REY-rich mud with moderate REY concentrations (ΣREY + Ce = 400–1,000 ppm) near the seafloor surface^2^ and the zones of greatest economic interest regarding ferromanganese crusts and nodules, including the central Pacific prime crust zone and Clarion–Clipperton Zone, respectively^33^. This finding supports the interpretation of IC1 as having enrichment in both biogenic Ca-phosphate and the hydrogenous component. Moreover, these high-IC1 areas overlap regions where O_2_ penetration may occur throughout the sediment column^34^. This implies that a sustained condition of higher redox potential might prevent diagenetic processes that release and remigrate Ce through decomposition of hydrogenous Mn oxides to maintain the intense IC1 signal throughout geologic time.

The strongest IC4 intensities, 9–10, were observed at DSDP Sites 597 and 74 in the eastern South Pacific (Fig. 3c). The deeper portion of Ocean Drilling Program (ODP) Sites 800 and 801 in the western North Pacific at present also showed high IC4 scores of 5–8, which plotted in the central North Pacific according to plate motion. When moderate values of 1–3 were included, IC4 effectively mimicked the distribution of REY-rich mud near the seafloor surface^2^ despite the fact that Fig. 3c is a synthetic image of different time slices during the Cenozoic. This suggests that the spatial distribution of the REY-controlling factor shown as IC4 did not change significantly throughout the Cenozoic.

IC7 indicates the influence of the hydrothermal component on the chemical composition of the sediment. Thus, the IC7 scores reflect the relative influence of the hydrothermal component and other dilutive components such as biogenic carbonate at each site. Despite vigorous present-day hydrothermal activity^35^, the sediments near the East Pacific Rise (EPR) in the eastern South Pacific showed relatively low IC7 scores (e.g. Sites 598–602; Fig. 3f). This is attributed to the significant dilution of the hydrothermal component by biogenic carbonate owing to water depth shallower than the CCD^19^. The water depth became greater than the CCD with time owing to plate migration, which resulted in a decreased dilution effect by the biogenic carbonate and increased relative influence of the hydrothermal component. This effect appears to be reflected in the increase of IC7 signals during the last 15 Myr at DSDP Sites 75 and 597 (Fig. 3f). It is noteworthy that the hydrothermal component reaches these sites located >2,000 km west of the EPR, which is consistent with data from the GEOTRACES cruise showing lateral westward transport of hydrothermal dissolved Fe and Mn several thousand kilometres from the present southern EPR^36^. The strongest IC7 signal during the Cenozoic appeared in the eastern North Pacific (Sites 37–39 and 1215; Fig. 3f). In contrast to the southern EPR sites, the water depths at these sites have been deeper than the CCD since the Eocene; thus, dilution of the hydrothermal component by biogenic carbonate has not occurred since that time (Fig. 3b; Supplementary Fig. S18). However, the IC7 signal that was maintained at a higher level than that in other areas, together with the Fe–Mn concentrations, showed a stepwise decrease to negative values during the Miocene (Fig. 3f; Supplementary Fig. S18). If plate migration and change in distance from the mid-ocean ridges caused this reduction, the hydrothermal deposition should have gradually and consistently decreased upward from the core bottom. Therefore, we consider the possibility that relatively rapid reduction can be attributed to the disappearance of the hydrothermal source owing to subduction of the ridge during the mid-Miocene^37^ (Supplementary Fig. S18). However, other regional factors such as changes in ocean currents resulting in migration of hydrothermal plumes could also affect the IC7 signal intensity.

### Rare-earth element geochemistry

To further clarify the geochemical interpretations of IC1 and IC4, REY-rich mud samples strongly influenced by either IC1 or IC4 were selected, and their REY-patterns normalised to the post-Archean average Australian shale^38^ were compared (Fig. 4). Because the variables analysed by ICA do not contain explicit information of each lanthanoid element and Y content, this approach can provide an additional geochemical constraint.

The bulk REY-patterns of representative high-IC4 sediments obtained from the eastern South Pacific and central North Pacific are characterised by distinct negative Ce anomalies, positive Y anomalies, and relative enrichment in heavy rare-earth to light rare-earth elements (Fig. 4g–l). These features are almost the same as those of biogenic Ca-phosphate^22^,^23^,^24^,^25^ (Fig. 4m), suggesting that the bulk-sediment REY compositions of high-IC4 sediments were predominantly controlled by highly REY-enriched biogenic Ca-phosphate.

The representative high-IC1 sediments are distributed in the central South Pacific, central North Pacific, and eastern Indian oceans. The Indian Ocean’s REY-rich mud contains no age-diagnostic fossils^3^ and is thus not shown in Fig. 3. In contrast to the case of high-IC4 sediments, the bulk REY patterns of high-IC1 sediments were characterised commonly by smaller negative Ce anomalies, almost no Y anomaly, and relatively flat REY patterns (Fig. 4a–f). These features can be reasonably explained^4^ by superimposing the REY pattern of slowly precipitating hydrogenous Fe–Mn oxide (δ–MnO_2_) showing pronounced positive Ce and negative Y anomalies (Fig. 4m) on that of biogenic Ca-phosphate in pelagic clay inheriting distinct negative Ce and positive Y anomalies from the seawater^22^,^23^,^24^,^25^ (Fig. 4m). It is noteworthy that the bulk REY abundance in IC1-type REY-rich mud also appeared to be largely attributed to biogenic Ca-phosphate, considering its very high REY concentration (ΣREY + Ce > 20,000 ppm)^25^ and negative Ce anomalies in the bulk REY compositions. Therefore, the characteristic data distribution in the IC space was confirmed by a different geochemical index using elements not explicitly included in the ICA calculation.

## Discussion

Despite the very high concentration of REY in biogenic Ca-phosphate, these elements are not incorporated into Ca-phosphate in significant amounts during the primary skeleton-forming process in living organisms^21^,^23^,^39^ (Fig. 4m). Therefore, the REY-enrichment process by biogenic Ca-phosphate in pelagic sediments has long been studied by a number of investigators^17^,^18^,^21^,^22^,^23^,^24^,^40^,^41^,^42^. Some have suggested that biogenic Ca-phosphate incorporates REY via pore water in the sediment column^23^,^40^. Others have argued that Ca-phosphate takes up REY from a variety of carrier phases (e.g. Fe–Mn-oxyhydroxides, pellets, and organic debris) that absorb REY from seawater and are readily decomposed at the sediment–water interface^17^,^22^,^41^,^42^. Although the complete process remains an open question, the characteristic REY patterns indicate that seawater is the ultimate source of the REY presently captured in biogenic Ca-phosphate^22^,^25^,^41^ (Fig. 4m).

Simple quantitative estimation in this study suggested the possibility that seawater can contain the flux of REY precipitation required to explain the observed very high REY concentrations (ΣREY + Ce > 1,000 ppm) in bulk REY-rich mud with a sedimentation rate less than ~0.5 m/Myr (Supplementary Fig. S19). Such circumstances may facilitate the concentration of REY in biogenic Ca-phosphate via diffusion of REY from seawater or the transfer of REY from original carriers to Ca-phosphate during the early diagenetic processes because of the prolonged exposure to seawater at the sediment surface and in the bioturbated and well-ventilated uppermost sediment layer^17^,^18^,^22^,^41^. In addition, biogenic Ca-phosphate enriched in REY might be stabilised through recrystallisation as insoluble apatite^17^. Moreover, the amount of Ca-phosphate in a unit volume of sediment also increases with a depression of the sedimentation rate^17^,^18^.

Hence, the low sedimentation rate is considered to be crucial for the formation of REY-rich mud. Actually, the spatiotemporal distribution of high-IC1, -IC4, and -IC7 muds overlapped with the oligotrophic North Pacific and South Pacific gyres and with water depths greater than the CCD, both of which prevented the fast accumulation of dilutive components with low REY content such as biogenic carbonate and silica. If we assume that deep-sea sediments can continuously acquire REY from the overlying deep-sea water prior to burial, the REY-enrichment in sediments can occur in a time scale of ~10^5^ years considering the sedimentation rate of <0.5 m/Myr with a typical thickness of ~0.1 m for the well-ventilated uppermost sediment layer^43^. This timescale of REY enrichment is significantly longer than that of global ocean circulation, which is ~10^3^ years^44^.

Both IC1 and IC4 indicated statistical independent geochemical features of pelagic red clay mainly composed of detrital aluminosilicates involving abundant Si, Al, Fe, Mg, and K without significant contributions of biogenic carbonate and silica. Fe and Mn of hydrothermal or hydrogenous origins could have also been incorporated without dilution in high-IC1 and high-IC4 sediments, resulting in an increase in the contents of these elements. In addition, in deposition slow enough to allow biogenic Ca-phosphate grains to concentrate REY and to accumulate significantly in the sediment, the P and REY contents of these sediments also increase concurrently. Although sediments enriched in biogenic Ca-phosphate contain several percent of Ca, the significant dilution effect of biogenic carbonate, which contains several tens of percent of Ca, generally creates negative trends in these ICs as a whole in the spaces of Ca and other elements, including REY. The difference between IC1 and IC4 is the prominence of hydrogenous δ–MnO_2_ precipitation that uptakes Ce preferentially via oxidation of Ce (III) to Ce (IV), which might be attributed to the variation in concentration of dissolved Fe and Mn, including colloids, in water masses^36^,^45^,^46^; the regional variation in supply of teeth/bone debris of marine vertebrates to the sediment; or differences in the sedimentation rate which may affect accumulation of the hydrogenous component.

In addition, an increase in the depositional flux of biogenic Ca-phosphate likely coincided with an increased supply of organic matter to the seafloor, although the sedimentation rate was likely held at a sufficiently lower level than those of calcareous/siliceous ooze and near-shore terrigenous detrital sediment. The increased deposition of organic matter might have caused relatively oxygen-poor (suboxic) conditions near the sediment surface and enhancement of the geochemical interactions between sediments and ferromanganese nodules during early diagenetic processes^17^,^47^, which partially explains the geographical overlap of REY-controlling IC signals and ferromanganese nodules on the seafloor. As the next step, ICA on a higher dimensional dataset including various transition metals involved in ferromanganese nodules, such as Co, Ni, Cu, and Zn, would likely extract another characteristic signature of the geochemical processes.

On the basis of the statistical analysis of the integrated dataset, we can also deduce regional features from a global perspective. Among all of the samples analysed in the present study, the highest concentration of REY, ~2,800 ppm, occurred at DSDP Site 596 in the central South Pacific Ocean. Site 596 is one of a few sites where the sediment column of pelagic clay throughout the Cenozoic was recovered almost completely. The downhole variation of total REY contents in bulk sediments at the comparable Site U1365^8^ drilled during Integrated Ocean Drilling Program Expedition 329 shows the essentially same profile. At Site 596, IC1-type REY-rich mud containing >2,000 ppm of ΣREY plus Ce was deposited during the Palaeogene (i.e. 66 to 23 Ma; Fig. 5a). In a previous work^48^, temporal variations in the relative mass fractions of inferred sediment end-member components at Site 596 were calculated by using the total inversion method. The profile of our IC1 scores at Site 596 almost correlates with both the calculated hydrogenous and phosphate components (Fig. 5b–d). This confirms that the commingled accumulation of these two components^18^ extracted as IC1 generated the very high-grade REY-rich mud in the South Pacific during the Palaeogene. The occurrence of such mud might be explained by the absence of the major source of terrigenous aeolian dust and volcanic ashes that represent a significant fraction of pelagic red clay at the site. During the Palaeogene, the Australian continent was located farther south that its present position (Fig. 5f), and its climate was substantially more humid^49^,^50^. Therefore, the supply of aeolian dust from the Australian continent to the South Pacific could have been much smaller than the present level^8^. This, combined with the extremely low surface productivity in the ultra-oligotrophic South Pacific Gyre, should have resulted in an extremely low sedimentation rate in the area during the Palaeogene.

Subsequently, the IC1 scores at Site 596 abruptly decreased at ~23 Ma, or the Palaeogene/Neogene boundary, and remained low during the Neogene. This feature is attributed to the increased fraction of detrital materials including terrigenous aeolian dust and the andesitic component from convergent-margin magmatic arcs since the Paleogene/Neogene boundary^48^ (Fig. 5e). The increased fraction of detrital and volcanic components enhanced the sedimentation rate, which in turn suppressed the REY enrichment in the bulk sediments. The increased flux of terrigenous materials during the Neogene can be attributed to the increased aridity on the Australian continent since the middle Miocene^8^,^48^,^49^,^50^,^51^. The northward and northwestward motions of the Indo–Australian and Pacific plates (Figs 3 and 5f), respectively, moved the Australian continent into the subtropical high-pressure belt, or the dry climatic zone, and pushed the site closer to the continental dust source. This may have resulted in the significantly increased supply of terrigenous materials to the site (Fig. 5f). Combined with the previous works showing the important linkage between global environmental changes and the influence of aeolian dust on deep-sea sediments^8^,^48^, the behaviour of REY-enrichment IC at this site suggests that Earth system dynamics such as climate change, geochemical cycles, and plate tectonics have intricately affected the sedimentation rate in the central South Pacific Ocean and may have contributed to the formation of high-grade REY-rich mud in the area over geologic time.

## Methods

### Samples and data

**Figure 1:**
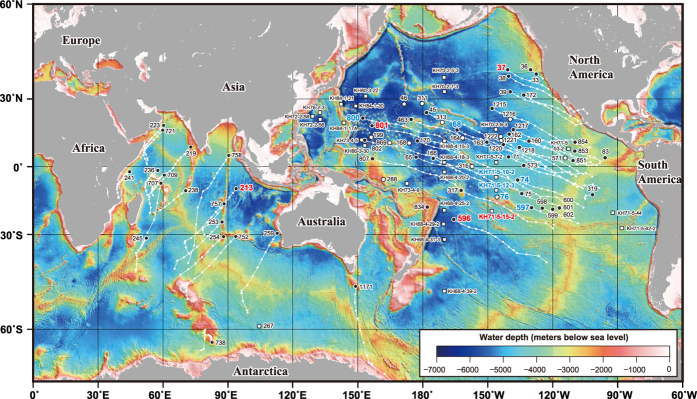
Locations of the sites used in this study. Circles represent Deep Sea Drilling Project/Ocean Drilling Program (DSDP/ODP) drilling sites, and squares indicate the University of Tokyo piston core sites. Sites filled in black are age-constrained and were thus used for reconstruction of the spatiotemporal distribution of independent components (ICs). White lines indicate tracks of each site, with tiny circles marking palaeopositions of each site in 5 Myr steps. Sites with red and blue labels indicate representative high-IC1 and high-IC4 sediments, respectively. Bathymetric data are from ETOPO2v2 (NOAA National Geophysical Data Center, 2006; https://www.ngdc.noaa.gov/mgg/global/etopo2.html). This map was created by using Generic Mapping Tools software ( https://www.soest.hawaii.edu/gmt/), Version 4.5.8 59, and GPlates software^30^,^31^ ( http://www.gplates.org), Version 1.2.0.

**Figure 2:**
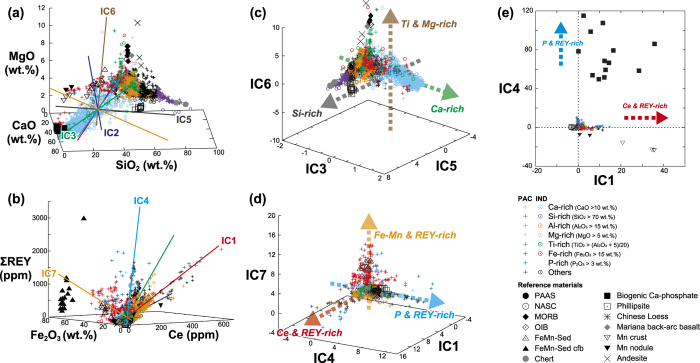
Compositional data of deep-sea sediments and extracted geochemical independent components (ICs). The ICs are projected in representative compositional subspaces of (**a**) SiO_2_–CaO–MgO and (**b**) Fe_2_O_3_–Ce–ΣREY (except Ce) as vectors showing specific directions along which the original chemical compositions changed. The label of each IC is placed along its positive axis. (**c**–**e**) show the data projected into IC subspaces. Dotted arrows indicate characteristic compositional changes along each IC axis. Sediment samples with specific compositions are colour coded as shown in the legend. No P-rich (P_2_O_5_ > 3 wt.%) sample was found in the Indian Ocean in the present work. Reference materials were not included in the dataset analysed by Independent Component Analysis (ICA) but were projected into IC spaces by using the same linear transformation as that for the sediment data, as shown in Supplementary Fig. S4. The data sources of the reference materials are compiled in Supplementary Fig. S2.

**Figure 3:**
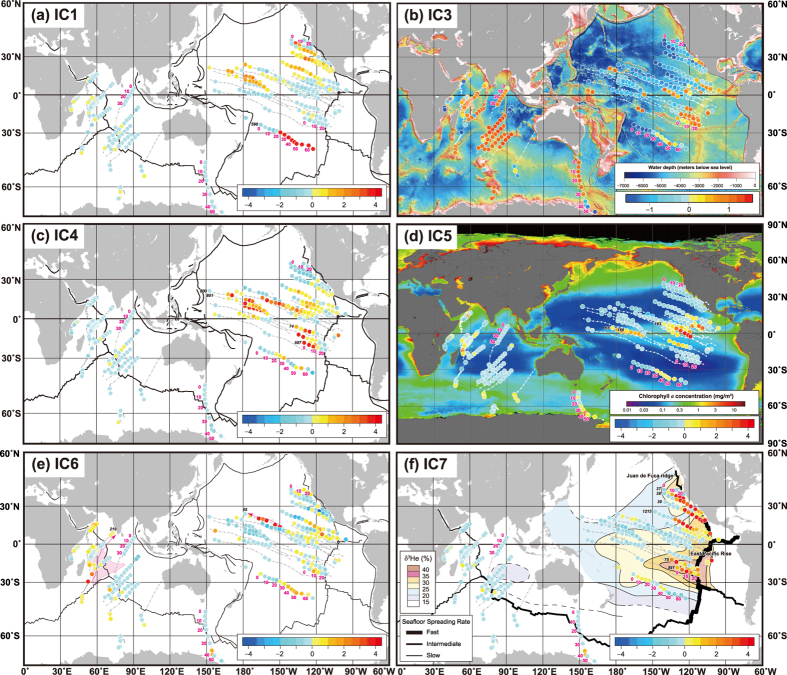
Spatiotemporal distributions of geochemical independent components (ICs) during the past 65 Myr: (**a**) IC1, (**b**) IC3, (**c)** IC4, (**d**) IC5, (**e**) IC6, and (**f**) IC7. IC scores, or the intensity of each IC, are the weighted mean values in each 5 Myr interval from the present to 65 Ma. Lines and tiny circles in grey or white indicate the tracks and palaeopositions of each site as shown in Fig. 1. The magenta numbers in the maps show the time interval in Ma at several representative sites. Several site numbers mentioned in the text are also shown in black. Modern ocean bathymetry (data are from ETOPO2v2; NOAA National Geophysical Data Center, 2006; https://www.ngdc.noaa.gov/mgg/global/etopo2.html) and modern global sea-surface chlorophyll a concentrations (courtesy of SeaWiFS Project, NASA Goddard Space Flight Center and ORBIMAGE; http://oceancolor.gsfc.nasa.gov/SeaWiFS/) are also shown in panels (**b**,**d**), respectively. In panel (**e**), dotted pink arrows indicate the tracks of Deep Sea Drilling Project (DSDP) Site 45 in the Pacific Ocean and that of DSDP Site 219 in the Indian Ocean. The palaeoposition of the Indian subcontinent at ~65 Ma is also shown. As indicators of hydrothermal plumes, the helium-3 anomalies (δ^3^He) of modern mid-depth seawater^35^ are also shown in (**f**). These maps were created by using the Generic Mapping Tools software ( https://www.soest.hawaii.edu/gmt/), Version 4.5.8 59, and GPlates software^30^,^31^ ( http://www.gplates.org), Version 1.2.0.

**Figure 4:**
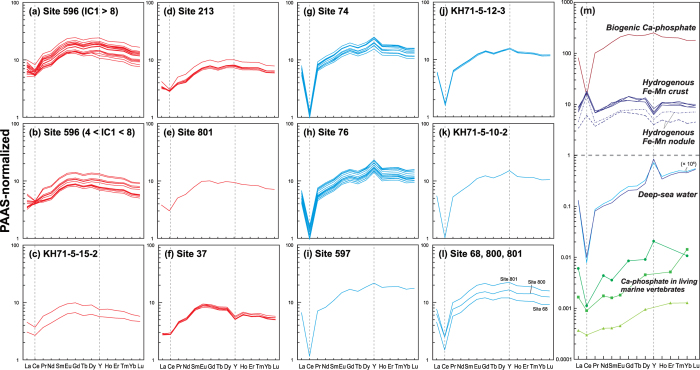
Average shale-normalised REY patterns of representative samples. PAAS^38^ was employed as the average shale. (**a**–**f**) correspond to high-IC1 samples, and (**g**–**l**) are high-IC4 samples. The REY patterns of biogenic Ca-phosphate in REY-rich mud^25^ and those in living marine vertebrates^23^,^39^, hydrogenous Fe–Mn oxides^33^,^60^, and deep-sea water in the western South Pacific Ocean^57^ and northeastern Indian Ocean^58^ are shown in (**m**) for comparison.

**Figure 5:**
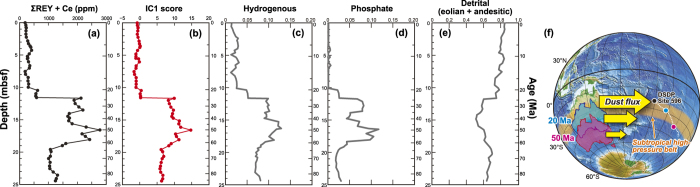
Temporal variations of REY content, IC1 score, and inferred relative mass fractions of sediment end-member components^48^ **at Deep Sea Drilling Project (DSDP) Site 596, central South Pacific.** (**a**) Bulk ΣREY plus Ce contents and (**b**) IC1 scores (this study). (**c**) Hydrogenous, (**d**) phosphate, and (**e**) detrital (sum of aeolian dust and andesitic materials from magmatic arc) components calculated by using the total inversion method^48^. Age was determined on the basis of a constant Co-flux model^48^. (**f**) Geographical relationship between the Australian continent and DSDP Site 596 during the Cenozoic. Blue and magenta colours indicate reconstructions at 20 Ma and 50 Ma, respectively. The orange band shows the present-day subtropical high-pressure belt. The sizes of the yellow arrows represent the inferred change in dust flux from the Australian continent associated with plate motion. This map was created by using the Generic Mapping Tools software ( https://www.soest.hawaii.edu/gmt/), Version 4.5.8 59, and GPlates software^30^,^31^ ( http://www.gplates.org), Version 1.2.0.

Kato *et al.*^2^ reported a huge geochemical dataset of 2,037 bulk sediment samples collected from 51 drill cores obtained by DSDP/ODP and 27 piston cores obtained by the Ocean Research Institute, University of Tokyo. In the present work, in order to completely characterise the bulk sediment composition, we remeasured the major element content by implementing X-ray fluorescence analysis, as well as loss on ignition, for all the samples of which major elements had been analysed using inductively coupled plasma mass spectrometry, as discussed in the Supplementary Information. By adding 268 samples from the studied sites in ref. 2 and four new sites in the Pacific Ocean (Supplementary Data S1) and by combining new data from 1,663 samples from the DSDP/ODP sites in the Indian Ocean^3^,^4^ (Supplementary Data S2), we constructed a comprehensive compositional dataset of 3,968 bulk sediment samples from 82 sites in the Pacific Ocean and 19 sites in the Indian Ocean, covering a large portion of the two oceans (Fig. 1). Data from piston core samples^2^ and details of the chemical analysis methods have been reported elsewhere^2^,^3^.

### Fundamentals of Independent Component Analysis

ICA was first applied to the field of geochemistry to decode mantle isotopic signals by Iwamori and Albarède^11^, who clearly demonstrated that ICA can successfully extract independent geochemical signals from the data structure. The principles of ICA have been concisely reviewed^11^,^12^, and the theoretical background and numerical methods have been described in detail^9^. The crucial point of ICA is to extract the non-Gaussianity inherent in the data structure. According to the central limit theorem, the sum of independent random variables becomes closer to a Gaussian variable than any of the original random variables. Conversely, when a sum of variables deviates maximally from a Gaussian distribution, it is equivalent to one of the independent source signals or ICs.

To apply ICA to the compositional data of deep-sea sediments, we assumed that the data included a mixture of geochemical components characterised by mutually independent signatures. Our ICA problem is the basic linear mixing model represented as follows, which is the same expression as that used in other traditional multivariate analytical methods such as PCA:


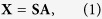


where **X** is the observed data matrix of which the rows and columns correspond to the individual samples and observed variables (i.e. elemental contents), respectively, **S** is an independent source matrix whose columns correspond to ICs, and **A** is a linear mixing matrix. In the ICA literature^9^, the model is usually explained by using the random variable vector, as discussed in the Supplementary Information. However, for consistency with our practical calculation^52^, we denote our ICA model by equation (1) using matrices, which is mathematically equivalent to that in the literature^9^. Both **S** and **A** are unknown and need to be estimated. First, the data matrix **X** is centred according to the mean of each variable. Next, the whitened data matrix **Z** is calculated by


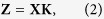


where **K** is the whitening matrix generated from the eigenvalues and eigenvectors of the covariance matrix of **X**, both of which can be obtained by using a basic PCA algorithm^9^. At that time, each column in **Z** has zero mean and unit variance, and any pair of new variables is mutually uncorrelated but not necessarily independent. Then, to make the variables statistically independent, the whitened axes are rotated until the projection of the whitened data on the axes gives a histogram, regarded as a probability density function along each axis, with maximum non-Gaussianity. Here, the estimated independent source matrix **S** is obtained by


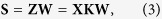


where **W**, known as the un-mixing matrix, is an orthogonal matrix for rotating the whitened data, **Z** and is numerically determined when an evaluation function of non-Gaussianity converges. As the evaluation function, we used negentropy *J*(*y*) approximated by


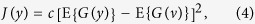


where *y* is a random variable of zero mean and unit variance (i.e. whitened and rotated data), *c* is an arbitrary constant, and *ν* is a Gaussian variable of zero mean and unit variance. The function G is defined as


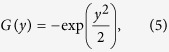


following refs 9,11. We employed the FastICA algorithm^9^ to implement the above numerical processes, and we utilised an open R package^52^ with some modifications to perform the calculation.

We used the contents of SiO_2_, TiO_2_, Al_2_O_3_, Fe_2_O_3_, MnO, MgO, CaO, K_2_O, P_2_O_5_, ΣREY, and Ce as variables in the observed data matrix **X**. The values of major element contents are expressed in weight percent; those of ΣREY and Ce are in parts per million. The difference in units does not affect the result of the ICA because the data are whitened to have unit variances for each variable. The number of ICs to be extracted is one of the important parameters for ICA^11^,^12^. Considering the balance of dimension reduction to exclude noise and retention of original information, we regarded the components that account for less than 1% of the total sample variance as unimportant signals, including noise. Although the statistical detection limit is often set at a somewhat higher level in common PCA or FA (e.g. 2% of the variance^8^), we selected 1% because ICA is expected to extract a signal of even small power when it contains clear non-Gaussianity. On the basis of this criterion, we extracted seven ICs in this study based on the eigenvalues obtained by PCA (Supplementary Table S3).

To evaluate the variability of the ICA results owing to random initial values given in the FastICA algorithm^9^ to estimate the matrix **W**, we performed 100 computational runs of ICA on the fixed data matrix **X** repeatedly (Supplementary Fig. S7). Moreover, to evaluate the robustness of the ICA results to data uncertainties such as analytical error^11^,^14^, we performed an additional 100 computational runs repeatedly by using synthetic perturbed datasets created by normal random variables for each run, assuming that 5 relative per cent of the mean value of each elemental content corresponds to conceivable analytical error (Supplementary Fig. S7).

### Rare-earth elements and yttrium flux estimation

Considering that seawater is the ultimate origin of REY in REY-rich mud, the formation of REY-rich mud should be controlled by the flux of REY from seawater to the sediment surface. Following a simple method^22^, we estimated the required REY flux for generating REY-rich mud on the basis of the results of the present study, and we compared the value with the REY-precipitation flux provided by the overlying water column. We used Nd as a representative of REY because this element has long been studied as a tracer of ocean circulation, and its residence time in the ocean appears to be the most reliable^53^,^54^.

The mass of Nd contained in one unit volume of sediment can be written as


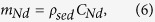


where* ρ*_*sed*_ is the dry bulk density of the sediment and *C*_*Nd*_ is the bulk content of Nd in the sediment. The regression line between the bulk Nd and total REY content *C*_*REY*_ in our data is


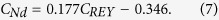


*C*_*Nd*_ and *C*_*REY*_ showed very good linearity, with R^2^ = 0.981 (*n* = 3,968). Substituting equation (7) for equation (6), the required flux *F* of Nd to explain the mass of Nd contained in one unit volume of sediment is described as


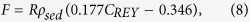


where *R* is the sedimentation rate.

Conversely, the mass of Nd contained in the water column from the sea surface to the seafloor is


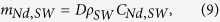


where *D* is the water depth, *ρ*_*SW*_ is the density of the seawater, and *C*_*Nd,SW*_ is the concentration of Nd in the seawater. Assuming that all of the Nd contained in the water column overlying the sediment of a unit area settled onto the seafloor within its residence time τ_*Nd*_ in the ocean^22^, the Nd-precipitation flux from the overlying seawater *F*_*SW*_ can be estimated by


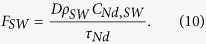


The requisite condition for the formation of REY-rich mud with a bulk total REY content *C*_*REY*_ is


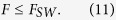


By substituting equations (8) and (10), equation (11) can be solved as


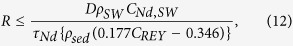


which indicates that the requirement for the formation of REY-rich mud is a sedimentation rate smaller than a particular value.

Supplementary Fig. S19 shows the relationship between the Nd precipitation flux and the sedimentation rate. We assumed *D* = 5,000 m, *ρ*_*sed*_ = 0.5 g/cm^3^ (Supplementary Table S4), *ρ*_*SW*_ = 1.03 g/cm^3^, and τ_*Nd*_ = 380–950 years^55^. In the present ocean, the concentrations of REY in the seawater are generally low in the surface and high in the deep ocean^56^,^57^,^58^. Here, we used the following averaged concentration of dissolved Nd through the water column based on ref. 56: *C*_*Nd,SW*_ = 3.54 × 10^−6^ ppm. Supplementary Fig. S19 shows that the condition for feeding REY-rich mud of relatively high total REY concentration (>1,000 ppm, including Ce) is *R *≤ ~ 0.5 m/Myr. Indeed, the sedimentation rates of typical REY-rich mud with high-IC1 or high-IC4 scores are generally less than 0.5 m/Myr (Supplementary Table S4). However, their values contain large uncertainty owing to the paucity of age-diagnostic fossils in the pelagic clay.

## Additional Information

**How to cite this article**: Yasukawa, K. *et al.* Tracking the spatiotemporal variations of statistically independent components involving enrichment of rare-earth elements in deep-sea sediments. *Sci. Rep.* **6**, 29603; doi: 10.1038/srep29603 (2016).

## Supplementary Material

Supplementary Information

Supplementary Dataset 1

Supplementary Dataset 2
