# Supplementary material for: Tracking the spatiotemporal variations of statistically independent components involving enrichment of rare-earth elements in deep-sea sediments
Source: Sci Rep. 2016 Jul 22;6:29603. doi: 10.1038/srep29603 (PMC4957207; doi:10.1038/srep29603)
Supplement: Supplementary Information [file srep29603-s1.pdf]

Tracking the spatiotemporal variations of statistically independent components involving enrichment of rare-earth elements in deep-sea sediments

Kazutaka Yasukawa[1,2], Kentaro Nakamura[1], Koichiro Fujinaga[2,1], Hikaru Iwamori[3,4] and Yasuhiro Kato[5,1,2,6]*

[1]Department of Systems Innovation, School of Engineering, The University of Tokyo, 7-3-1 Hongo, Bunkyo-ku, Tokyo 113-8656, Japan

[2]Ocean Resources Research Center for Next Generation, Chiba Institute of Technology, 2-17-1 Tsudanuma, Narashino, Chiba 275-0016, Japan

[3]Department of Solid Earth Geochemistry, Japan Agency for Marine-Earth Science and Technology (JAMSTEC), 2-15 Natsushima-cho, Yokosuka, Kanagawa 237-0061, Japan

[4]Department of Earth and Planetary Sciences, Graduate School of Science and Engineering, Tokyo Institute of Technology, 2-12-1 Oo-Okayama, Meguro-ku, Tokyo 152-8550, Japan

[5]Frontier Research Center for Energy and Resources, School of Engineering, The University of Tokyo, 7-3-1 Hongo, Bunkyo-ku, Tokyo 113-8656, Japan

[6]Research and Development Center for Submarine Resources, Japan Agency for Marine-Earth Science and Technology (JAMSTEC), 2-15 Natsushima-cho, Yokosuka, Kanagawa 237-0061, Japan

*Corresponding author: Yasuhiro Kato

Frontier Research Center for Energy and Resources, School of Engineering, The University of Tokyo, 7-3-1 Hongo, Bunkyo-ku, Tokyo 113-8656, Japan

Telephone: +81-3-5841-7022; email: ykato@sys.t.u-tokyo.ac.jp

Mathematical formulation of the Independent Component Analysis model

In the literature of Independent Component Analysis (ICA; Ref. S1), the mathematical model is usually formulated as

$$\mathbf{x} = \mathbf{A}\mathbf{s}, \quad \text{(S1)}$$

where **x** is the column vector whose *i*-th element is an observed random variable x_i (i = 1, 2, 3, ..., m, where m is the number of observed variables), **s** is the column vector whose *j*-th element is an independent random variable s_j (i.e. independent component, IC; j = 1, 2, 3, ..., r, where r is the number of ICs), and **A** is the $m \times r$ mixing matrix. In general, m and r may not be equal, and r should not be larger than m. Equation (S1) indicates that the observed data can be expressed by a linear combination of the independent source components, or the compositional data can be plotted in subspaces spanned by the new base vector **s**.

Practically, however, **x** contains the index of the time or sample (Ref. S1) and thus can be written as $x_i(t)$ (t = 1, 2, 3..., n, where n is the number of samples). Therefore, in our problem, **x** is equivalent to the $m \times n$ observed data matrix whose rows and columns correspond to the m variables and n samples, respectively, which can be denoted by the transposed matrix $\mathbf{X}^\mathrm{T}$. The superscript T denotes the transpose, and **X** is the same as the $n \times m$ observed data matrix defined in 'Fundamentals of ICA' in the Methods section. In the same manner, **s** can be written as the $r \times n$ independent source matrix $\mathbf{S}^\mathrm{T}$ because t is the common index between **x** and **s** (Ref. S1). In addition, if we arbitrarily rewrite the mixing matrix **A** in equation (S1) as $\mathbf{A}^\mathrm{T}$, where $\mathbf{A}^\mathrm{T}$ is also an $m \times r$ matrix because this is a merely notational change, the formulation of equation (S1) can be written as

$$\mathbf{X}^\mathrm{T} = \mathbf{A}^\mathrm{T}\mathbf{S}^\mathrm{T} \quad \text{(S2)}$$

By transposing both sides of equation (S2), we obtain

$$\mathbf{X} = \mathbf{S}\mathbf{A}, \quad \text{(S3)}$$

where **X** is the $n \times m$ observed data matrix, **S** is the $n \times r$ independent source matrix, and **A** is the $r \times m$ mixing matrix (Refs. S2, S3).

In the open R package that we employed to perform ICA (Ref. S2), the observed data matrix **X** is assumed to have the samples in its rows and the variables in its columns (i.e. an $n \times m$ matrix). Indeed, actual data are often organized as a matrix whose rows correspond to individual samples, and the ICA model for such data was represented by the same equation as (S3) (e.g. Ref. S3). Therefore, for the convenience of the potential users of the package based on this paper, we denoted our ICA model by equation (S3), as discussed in 'Fundamentals of ICA' in the Methods section.

Definitions of independent component scores and independent component loadings

We defined IC scores here as the coordinate values of the sample data in the IC space, which represent the intensities of each independent source signal in each sample. The IC score of the *j*-th element in the *i*-th sample corresponds to the (*i*, *j*) component of the estimated independent source matrix **S**.

We defined IC loadings here as the relative contributions of each element to the individual ICs, which correspond to the slope of each IC vector projected in the compositional space. In our ICA model, as given in the preceding section, the loadings of the *i*-th IC correspond to the *i*-th row of the estimated mixing matrix **A**. However, the absolute values of the contents and their variations differed significantly among elements. The CaO content varied from 0 to ~56%, whereas the Ce content varied from 0 to ~660 ppm, which makes it difficult to evaluate the contribution of each element to each IC at a glance. Therefore, in the present paper, to clearly visualize the influence of each element to each IC, we show the relative loading values rather than the raw loading values (Supplementary Fig. S6). Because the ICs do not have specific norms, the values of the relative loadings can be expressed in arbitrary units. A relative loading of the *j*-th element for the *i*-th IC can be obtained by dividing a_{ij}, or the (*i*, *j*) component of **A**, by the sample standard deviation of the *j*-th element.

The meaning of positive and negative signs in Independent Component Analysis results

The positive and negative loadings represent the elements that increase and decrease accompanying the unit increase of each IC score. In fact, ICA contains the ambiguity of the sign of each IC. In the ICA model, because both the independent source matrix **S** and the mixing matrix **A** are estimated at the same time, we can multiply the *i*-th independent component (i.e. the *i*-th column of **S**) and the corresponding *i*-th row of **A** by −1 simultaneously without affecting the analysis (Refs. S1 and S4). Therefore, we can arbitrarily choose the signs of the IC scores and loadings. Here, for an intuitive description of the geochemical ICs, we adjusted the signs of the IC scores and loadings as the characteristic elements increase when the IC score positively increases. This adjustment never affects the essential meaning of the ICA result.

Checking the statistical robustness of the independent components

1. Effect of outliers in the dataset

In this study, to minimize the undesirable influence of outliers or specific

sites/lithologies/samples, we chose the exponential function as the evaluation function of non-Gaussianity (i.e. G(y) in 'Fundamentals of ICA' in the Methods section) because it reflects the characteristic structure around the centre (mean) of the data more intensely than other proposed functions such as kurtosis, and it theoretically gives the most robust results theoretically (Refs. S1 and S5).

Here, we checked the robustness of our ICA result by using a limited dataset, excluding the samples with high values for each IC (greater than 3) in the original result. Because each IC is constrained to have unit variance in the fast ICA algorithm (Ref. S1), an IC score of 1 corresponds to 1 standard deviation (1σ) along each IC. Thus, samples having IC scores greater than 3 constitute the long tails outside the 3σ range in the IC score distributions that have strong non-Gaussianity such as large skewness and kurtosis (Supplementary Fig. S5). IC3 ranges from –1.7 to 1.3 and does not show a long-tailed distribution like other ICs (Supplementary Fig. S5). Therefore, no outlier was considered for IC3.

The ICA result with no outliers (n = 3,492) was fundamentally the same as the original result (Supplementary Fig. S8 and S9). To confirm this, we further calculated the projection of the excluded 'outlier' samples into the IC spaces by using the whitening matrix and the un-mixing matrix estimated from the dataset excluding high-IC samples (Supplementary Fig. S10). The projected data distributions of all samples were almost identical to the original ICA result derived from all samples (Supplementary Fig. S4), although only IC6 showed a slightly oblique feature compared with the original result. In general, this test confirmed that the geochemical ICs derived from all data certainly reflect the fundamental structure in the dataset and are not artefacts generated by a small number of outliers.

2. Effect of the difference in sample subsets between the Pacific and Indian oceans

Here, we evaluated the change in ICA results change when each basin is treated as separate populations in the ICA. The ICA result using only the Pacific Ocean samples extracted almost the same components as the original result (Supplementary Figs. S11 and S12), although the loading values changed slightly. Thus, the slopes of IC vectors projected in the compositional space differed slightly from the original result.

On the contrary, the ICA result using only the Indian Ocean samples showed partly different features (Supplementary Figs. S13 and S14). The ICs corresponding to the original IC1, IC3, IC5, IC6, and IC7 were extracted, although the loading of Mn in IC7 showed a somewhat smaller value than those in the hydrothermal IC in the original and Pacific Ocean results. The IC4 showing Ca-phosphate-enrichment was not extracted from the Indian Ocean

subset because the rare-earth elements and yttrium (REY)-rich mud samples in the Indian Ocean are predominantly IC1-type mud in our dataset. Alternatively, another IC assigned as new IC4 in the Indian Ocean result was extracted which showed a concurrent increase in Si, Ti, Al, Fe, Mg, and K (Supplementary Fig. S14). This IC signal appeared to be strong in the sites near the continent (e.g. Site 241 near the eastern coast of Africa, Site 223 off the Arabian peninsula, and Site 1171 south of Australia; Fig. 1 in the main text). This finding suggests that the IC corresponds to a terrigenous detrital component, although its contribution appears to be very minor in the entire dataset. The remaining ambiguous IC was assigned as IC2.

Although partly different components were extracted, the main structures of the data appeared to be common to data subsets from the two ocean basins. Thus, we discussed the general mechanism of REY-enrichment on the basis of the entire sample set in the main text.

3. Effect of the recalculation of raw data of major elements

In our dataset, the major element contents were determined by X-ray fluorescence (XRF) analysis, as discussed in the Methods section. Then, the total contents of the major elements (i.e. SiO_2, TiO_2, Al_2O_3, Fe_2O_3, MnO, MgO, CaO, Na_2O, K_2O, and P_2O_5) and loss on ignition (LOI) were normalized to 100 wt.% (Supplementary Data S1 and S2), although Na_2O and LOI were not included in the dataset analysed by ICA because of the effect of sea salt and the ambiguity of the origin, respectively.

To check the influence of the recalculation of raw data of the major elements, we performed ICA on the dataset by using XRF raw data of the nine major elements before normalizing to 100%, including LOI (Supplementary Figs. S15 and S16). The test resulted in essentially the same figures as the original ICA result discussed in the main text (Supplementary Figs. S4 and S6).

4. Additional ICA test on a sample subset without significant dilutions by using selective variables

We performed ICA on the sample subset, excluding high-Ca (CaO > 10 wt.%) and high-Si (SiO_2 > 70 wt.%), with the selective elements of Fe_2O_3, MnO, CaO, and P_2O_5 and all of the separated REY elements. As described below, the additional ICA resulted in extraction of three key ICs. The new IC1, IC2, and IC3 corresponded well to the original IC7, IC4, and IC1, respectively. It should be noted that even if the number of extracted ICs was increased, the new three key ICs were always extracted and essentially never changed. Therefore, we consider that these are inherent features of REY-rich mud characterized by the original IC1, IC4, and IC7. The result of the additional ICA is shown in Supplementary Figure S17, which indicates the

relative loadings of each IC in the cases in which the numbers of extracted ICs were 3 and 4.

The new IC1 largely contributes to Fe_2O_3 and MnO, and the samples with high new IC1 scores are from Sites 38, 39, and 1215 in the eastern North Pacific. These factors indicate that the new IC1 corresponds to the original IC7.

The new IC2 largely contributes to P_2O_5 and all REY elements except Ce, indicating the influence of biogenic Ca-phosphate. The samples with high new IC2 scores are from the eastern South Pacific and central North Pacific (Sites 74, 75, 76, 319, 597, and 800). These results indicate that the new IC2 corresponds to the original IC4.

The new IC3 distinctly contributes to the Ce content, indicating the influence of hydrogenous Mn oxides, and to the P_2O_5 content. This indicates that biogenic Ca-phosphate also affects the compositional change represented by the new IC3. The samples with high new IC3 scores are from Site 596 in the central South Pacific and Site 213 in the eastern Indian Ocean. These results led us to consider that the new IC3 corresponds to the original IC1.

When the number of ICs was increased to 4, the above three ICs were extracted again with no essential changes. This confirms that these three ICs extract the fundamental features inherent in the data structure. The additionally extracted IC4 has relatively large loadings of Fe_2O_3 and MnO, suggesting the influence of the hydrothermal component. This IC also largely contributes to P_2O_5 content, which implies the influence of biogenic Ca-phosphate. In addition, the relatively large Y loading compared with the other REEs, together with negative Ce loading, is characteristic of this new IC4. The samples with high IC scores regarding the new IC4 are from Sites 75, 319, and 597 in the eastern South Pacific, which showed intense signals in both original IC4 (i.e. biogenic Ca-phosphate) and IC7 (i.e. hydrothermal). The additional IC4 might reflect the influence of a relatively minor effect superimposed on the fundamental features, such as a diagenetic process. However, for more detailed and reliable discussion, various trace elements should be incorporated in the dataset.

Remeasurement of major element contents in previous data by X-ray fluorescence

In our inductively coupled plasma mass spectrometry (ICP-MS) analysis, bulk sediment samples were dissolved by HNO_3–HF–$HClO_4$ digestion, and the sample solutions were finally evaporated until dry. During this process, silicate minerals were digested by HF, and Si was lost as SiF_4 through evaporation. Therefore, ICP-MS analysis of acid-digested samples cannot determine the Si content. In addition, the LOI content was not analysed. The SiO_2 content in the previously published data by Ref. S6 was estimated by using the analysed contents of other

major element oxides and was assumed to have a constant value of $H_2O + CO_2$. In this study, to construct a complete dataset of uniform quality, we reanalysed all of the major elemental contents by using XRF, and we measured the LOI for all samples used in the ICA.

Comparison with previous studies

In a previous work (Ref. S6), Kato et al. reported two apparent trends in the Fe_2O_3–REY content diagram. The first was an Fe-rich trend associated with hydrothermal Fe-oxyhydroxides, and the second was a phillipsite trend characterized by relatively low Fe content and higher enrichment in REY. Phillipsite is a type of zeolite mineral that typically occurs in pelagic areas with very low sedimentation rates (Ref. S7). Determination of the phillipsite trend was based on previous ICA results in which extracted REY-enriched components showed features similar to phillipsite in major element composition (i.e. relatively low Fe and high Al contents). However, a recent direct measurement of REY abundance in phillipsite grains demonstrated that the grains themselves do not show REY enrichment (Ref. S8). Hence, we considered the possibility that a low sedimentation rate enhances both the enrichment of REY and the formation of phillipsite in pelagic sediments independently, resulting in a spurious correlation between REY content and phillipsite abundance (Refs. S9 and S10). Indeed, typical REY-rich mud with relatively high REY content is generally described as zeolitic clay; thus, the REY-enriched IC that showed an Al-rich trend in the previous study should have extracted the composite feature of pelagic clay abundant in phillipsite that also contains other aluminosilicates, Fe–Mn (oxyhydr)oxides, and REY-enriched biogenic Ca-phosphate.

Age models

Age models were constructed for each site on the basis of previously published biostratigraphic data. We assigned numerical ages to our samples by linear interpolation between the closest age-diagnostic events such as first/last occurrence of key fossils (e.g. calcareous nannofossils, foraminifera, and radiolaria) by using the geologic time scale (Ref. S11). For the Pacific Ocean sites, we referred to a compilation of numerical ages for the Neogene (Ref. S12). Data for the Palaeogene were compiled from the Deep Sea Drilling Project/Ocean Drilling Program (DSDP/ODP) Initial Reports volumes for each site (Supplementary Data S1). Moreover, we used the age models for DSDP Site 596 (Ref. S13) and for sites of ODP Leg 199 (Ref. S14). For the Indian Ocean sites, we compiled available biostratigraphic data from the Initial Reports volumes for each site (Supplementary Data S2).

Reconstruction of spatiotemporal distribution of independent component scores

Reconstruction of palaeogeography and the tracks of each site from the present day to 65 Ma was achieved by using GPlates (Refs. S15 and S16) in steps of 5 Myr. On the palaeomap of each time slice, the IC scores were plotted on the palaeopositions of each site as age-weighted mean values. We calculated the weighted mean of IC scores $\overline{s}_j$ at time slice T_i as

$$d_j = \left|T_j - t_j\right|, \tag{S4}$$

$$w_j = \frac{1}{d_j}, \tag{S5}$$

$$\overline{s}_j = \frac{\sum_j \left(w_j * s_j\right)}{\sum_j w_j}, \tag{S6}$$

where t_i is an assigned age of sample j, d_j is the age deviation between the time slice i and sample j, w_j is the weight of sample j, and s_j is the IC score of sample j. Equations (S4) to (S6) indicate that the closer in age a sample is to each time slice, the larger the weight of the sample. The values of T_i were 0 to 65 Ma in 5 Myr steps, and the weighted means were calculated by using samples within intervals of T_i ± 2.5 Ma except for 0 Ma and 65 Ma, in which the intervals were given as 0–2.5 Ma and 62.5–65 Ma, respectively.

Supplementary References

S1. Hyvärinen, A., Karhunen, J. & Oja, E. *Independent Component Analysis*. John Wiley & Sons, New York, U.S., 481 pp (2001).

S2. Marchini, J. L., Heaton, C. & Ripley, B. D. *Package 'fastICA' Reference manual.* (2013) http://cran.r-project.org/web/packages/fastICA/index.html (Accessed: 27th March 2016).

S3. Liebermeister, W. Linear modes of gene expression determined by independent component analysis. *Bioinformatics* **18,** 51–60 (2002).

S4. Iwamori, H., Albarède, F. & Nakamura, H. Global structure of mantle isotopic heterogeneity and its implications for mantle differentiation and convection. *Earth Planet. Sci. Lett.* **289,** 339–351 (2010).

S5. Iwamori, H. & Albarède, F. Decoupled isotopic record of ridge and subduction zone processes in oceanic basalts by independent component analysis. *Geochem. Geophys. Geosyst.* **9,** Q04033 (2008). doi:10.1029/2007GC001753.

S6. Kato, Y. *et al.* Deep-sea mud in the Pacific Ocean as a potential resource for rare-earth elements. *Nat. Geosci.* **4,** 535–539 (2011).

S7. Stonecipher, S. A. Origin, distribution and diagenesis of phillipsite and clinoptilolite in deep-sea sediments. *Chem. Geol.* **17,** 307–318 (1976).

S8. Kon, Y. *et al.* Geochemical characteristics of apatite in heavy REE-rich deep-sea mud from Minamitorishima area, southeastern Japan. *Resour. Geol.* **64**, 47–57 (2014).

S9. Yasukawa, K. *et al.* Geochemistry and mineralogy of REY-rich mud in the eastern Indian Ocean. *J. Asian Earth Sci.* **93,** 25–36 (2014).

S10. Yasukawa, K. *et al.* Rare-earth, major, and trace element geochemistry of deep-sea sediments in the Indian Ocean: Implications for the potential distribution of REY-rich mud in the Indian Ocean. *Geochem. J.* **49,** 621–635 (2015).

S11. Gradstein, F. M., Ogg, J. G., Schmitz, M. D. & Ogg, G. M. *The Geologic Time Scale 2012*. Elsevier, Oxford, UK. 1144 pp (2012).

S12. Lyle, M. Neogene carbonate burial in the Pacific Ocean. *Paleoceanography* **18,** 1059 (2003). doi:10.1029/2002PA000777.

S13. Zhou, L. & Kyte, F. T. Sedimentation history of the south Pacific pelagic clay province over the last 85 million years inferred from the geochemistry of deep sea drilling project hole 596. *Paleoceanography* **7,** 441–465 (1992).

S14. Pälike, H. *et al.* A Cenozoic record of the equatorial Pacific carbonate compensation depth. *Nature* **488,** 609–615 (2012).

S15. Müller, R. D., Sdrolias, M., Gaina, C. & Roest, W. R. Age, spreading rates and spreading

asymmetry of the world's ocean crust. *Geochem. Geophys. Geosyst.* **9,** Q04006 (2008). doi:10.1029/2007GC001743.

S16. Boyden, J. A. *et al.* Next-generation plate-tectonic reconstructions using GPlates. In *Geoinformatics: Cyberinfrastructure for the Solid Earth Sciences.* (eds. Keller, G. R. and Baru, C.), 95–113 (2011). Cambridge University Press.

S17. Taylor S. R. & McLennan, S. M. *The continental crust: its composition and evolution.* Blackwell Scientific Publications, Oxford, 312 pp (1985).

S18. Gromet, L. P., Dymek, R. F., Haskin, L. A. & Korotev, R. L. The 'North American shale composite': its compilation, major and trace element characteristics. *Geochim. Cosmochim. Acta* **48,** 2469–2482 (1984).

S19. Goldstein, S. J. & Jacobsen, S. B. Rare earth elements in river waters. *Earth Planet. Sci. Lett.* **89,** 35–47 (1988).

S20. Jenner, F. E. & O'Neill, H. S. C. Analysis of 60 elements in 616 ocean floor basaltic glasses. *Geochem. Geophys. Geosyst.* **13,** Q02005 (2012). doi:10.1029/2011GC004009.

S21. Garcia, M. O., Jorgenson, B. A. & Mahoney, J. J. An evaluation of temporal geochemical evolution of Loihi Summit lavas: Results from *Alvin* submersible dives. *J. Geophys. Res.* **98,** 537–550 (1993).

S22. Jarvis, I. Geochemistry and origin of Eocene–Oligocene metalliferous sediments from the central equatorial Pacific: Deep Sea Drilling Project Site 573 and Site 574. *In* Mayer, L., Theyer, F., Barron, J. A., Dunn, D. A., Handyside, T., Hills, S., Jarvis, I., Nigrini, C. A., Pisias, N. C., Pujos, A., Saito, T., Stout, P., Thomas, E., Weinreich, N. and Wilkens, R. H. *Initial Reports of the Deep Sea Drilling Project*, vol **85,** U.S. Government Printing Office, Washington, 781–804 (1985).

S23. Barrett, T. J., Taylor, P. N. & Lugowski, J. Metalliferous sediments from DSDP Leg 92: the East Pacific rise transect. *Geochim. Cosmochim. Acta* **51,** 2241–2253 (1987).

S24. Barrett, T. J. & Jarvis, I. Rare-earth element geochemistry of metalliferous sediments from DSDP Leg 92: the East Pacific rise transect. *Chem. Geol.* **67,** 243–259 (1988).

S25. Plank, T. & Langmuir, C. H. The chemical composition of subducting sediment and its consequences for the crust and mantle. *Chem. Geol.* **145,** 325–394 (1998).

S26. Gallet, S., Jahn, B. & Torii, M. Geochemical characterization of the Luochuan loess–paleosol sequence, China, and paleoclimatic implications. *Chem. Geol.* **133,** 67–88 (1996).

S27. Hein, J. R., Mizell, K., Koschinsky, A. & Conrad, T. A. Deep-ocean mineral deposits as a source of critical metals for high- and green-technology applications: comparison with land-based resources. *Ore Geol. Rev.* **51,** 1–14 (2013).

S28. Kelemen, P. B., Hanghøj, K. & Greene, A. R. One view of the geochemistry of subduction-related magmatic arcs, with an emphasis on primitive andesite and lower crust. *In* Holland, H. D. and Turekian, K. K. eds. *Treatise on Geochemistry* Vol. **3,** 593–659 (2003).

S29. Müller, R. D., Sdrolias, M., Gaina, C., Steinberger, B. & Heine, C. Long-term sea-level fluctuations driven by ocean basin dynamics. *Science* **319,** 1357–1362 (2008).

S30. Wessel, P. & Smith, W. H. F. New, improved version of Generic Mapping Tools released. *Eos Trans. Amer. Geophys. U.* **79,** 579 (1998).

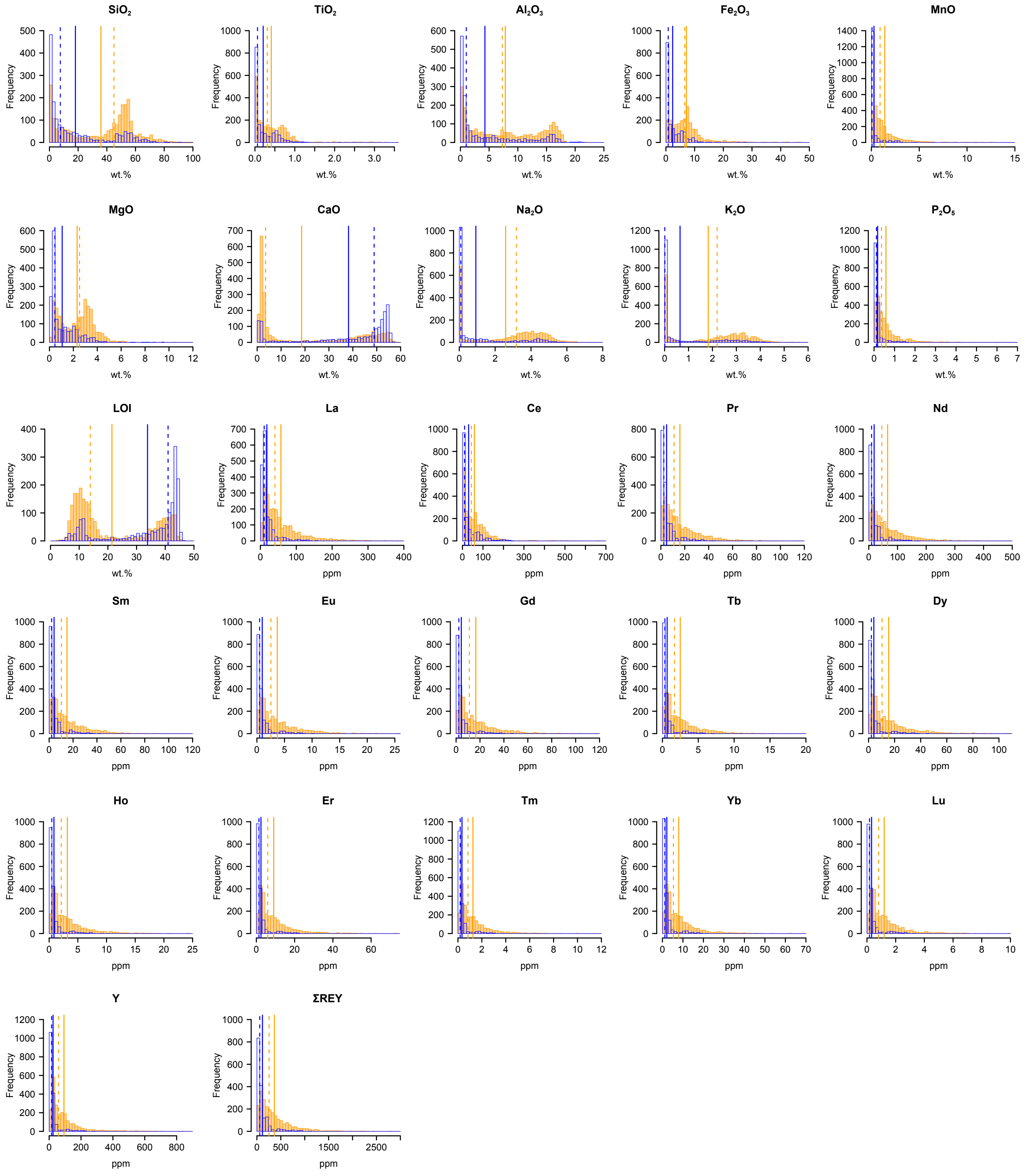

Supplementary Figure S1. Histograms of elemental content in the bulk sediment samples from the Pacific (orange) and Indian (blue) oceans. Vertical solid and dashed lines represent the mean and median of each data distribution, respectively.

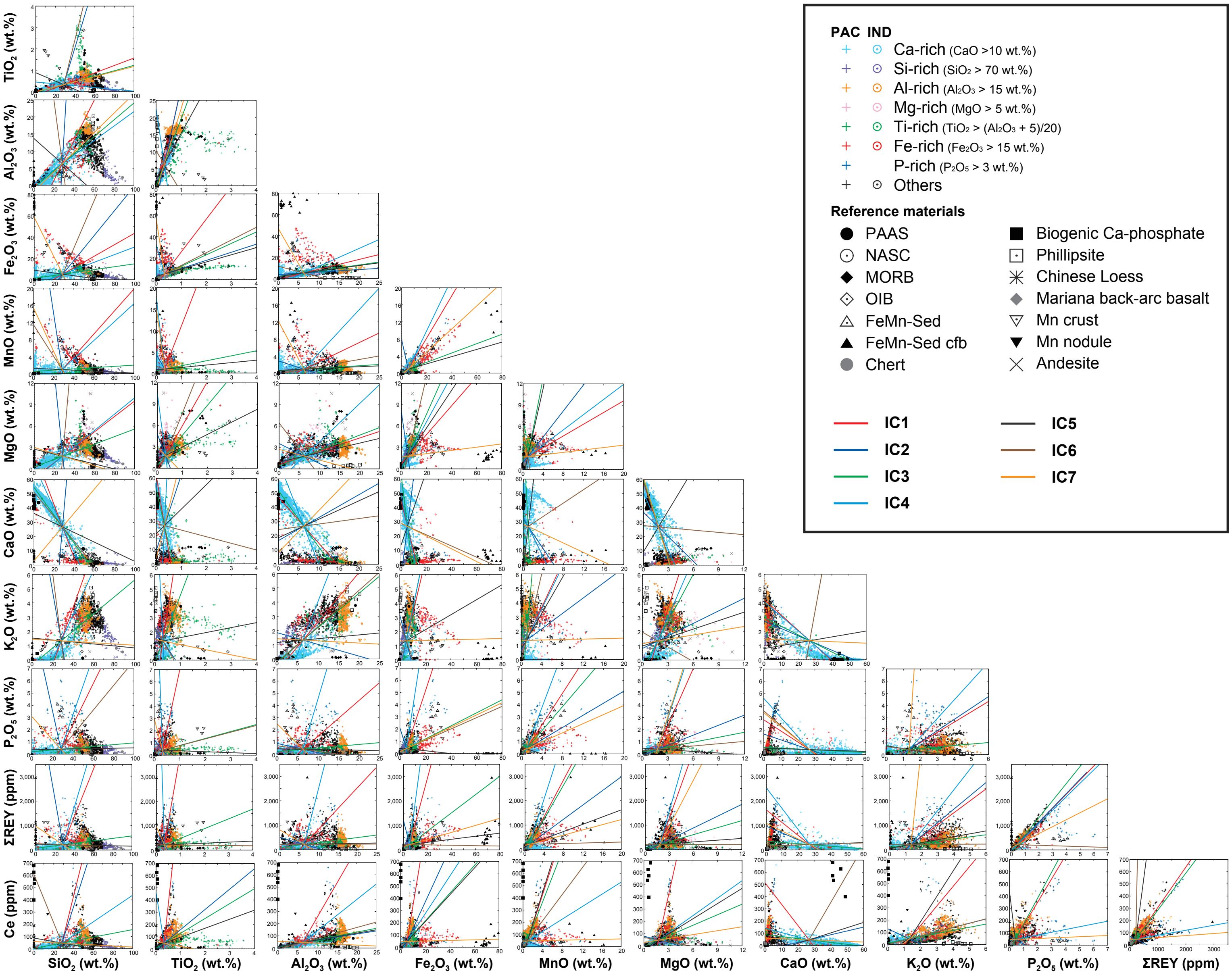

Supplementary Figure S2. Two-dimensional projection diagrams for the bulk chemical compositions and IC vectors extracted from the compositional dataset of deep-sea sediments in the Pacific and Indian oceans. Sediment samples with specific compositions are color-coded as shown in the legend. No P-rich ($P_2O_5 > 3$ wt.%) sample was found in the Indian Ocean in the present work. For comparison, several potential end-members in composition of deep-sea sediments were also plotted as reference materials. The data sources of the reference materials are as follows. PAAS: Ref. S17; North American shale composite (NASC): Refs. S18 and S19; mid-ocean ridge basalt (MORB) and Mariana back-arc basin basalt: Ref. S20; ocean island basalt (Hawaiian islands): Ref. S21; hydrothermal Fe–Mn-rich sediment: Ref. S22; hydrothermal Fe–Mn-rich sediment (carbonate-free basis, cfb): Refs. S23 and S24; chert (western North Pacific): Ref. S25; Chinese loess: Ref. S26; Mn crusts and nodules: Ref. S27; andesite: Ref. S28; and biogenic Ca-phosphate and phillipsite: Takaya et al. (unpublished data).

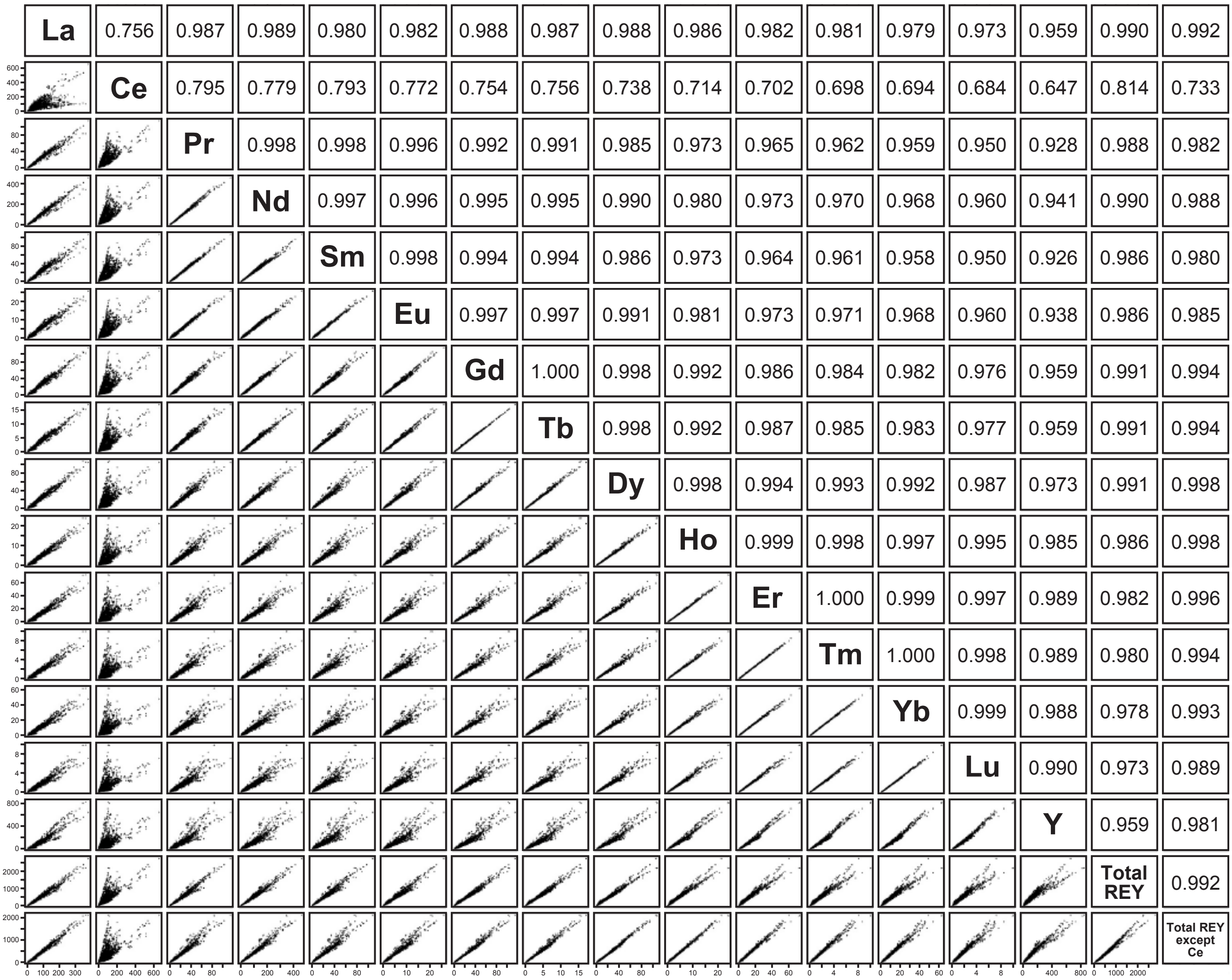

Supplementary Figure S3. A scatter plot matrix of all samples regarding individual REY and total REY contents. The diagonal components are the variables. The upper panels in the matrix represent correlation coefficients. The lower panels show 2-dimensional scatter plots of each pair of rare-earth elements. "Total REY" represents a sum of all REY elements including Ce. Note that correlations between Ce and other individual REY element (Pearson correlation coefficient $r = 0.647–0.795$) are distinctly weaker than those in any pair of individual REY except Ce ($r = 0.926–1.000$, generally $r > 0.95$), demonstrating that the geochemical behavior of Ce differs from that of other REY elements.

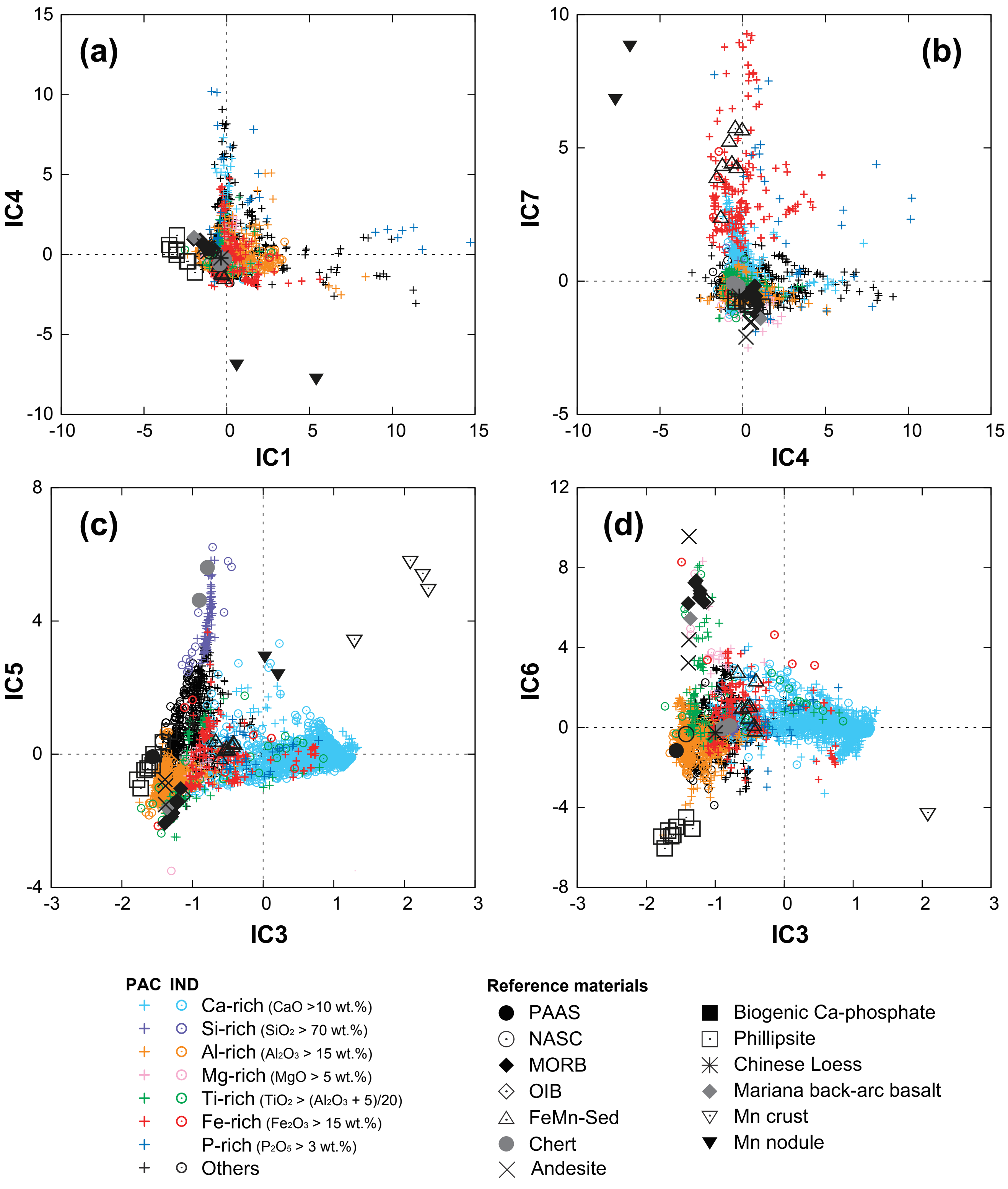

Supplementary Figure S4. Data distribution in selected 2-dimensional IC spaces. **(a)** IC1 vs. IC4, **(b)** IC4 vs. IC7, **(c)** IC3 vs. IC5, and **(d)** IC3 vs. IC6. Note that the reference materials were not included in a data set analyzed by ICA, but were merely projected into IC spaces by applying the same linear transformation as that estimated from samples by ICA. The approximate IC scores of the reference materials $\mathbf{S}_{rm}$ are calculated by multiplying their compositional data matrix $\mathbf{X}_{rm}$ by the whitening matrix $\mathbf{K}$ and the un-mixing matrix $\mathbf{W}$ estimated from the samples (i.e. $\mathbf{S}_{rm} = \mathbf{X}_{rm}\mathbf{K}\mathbf{W}$).

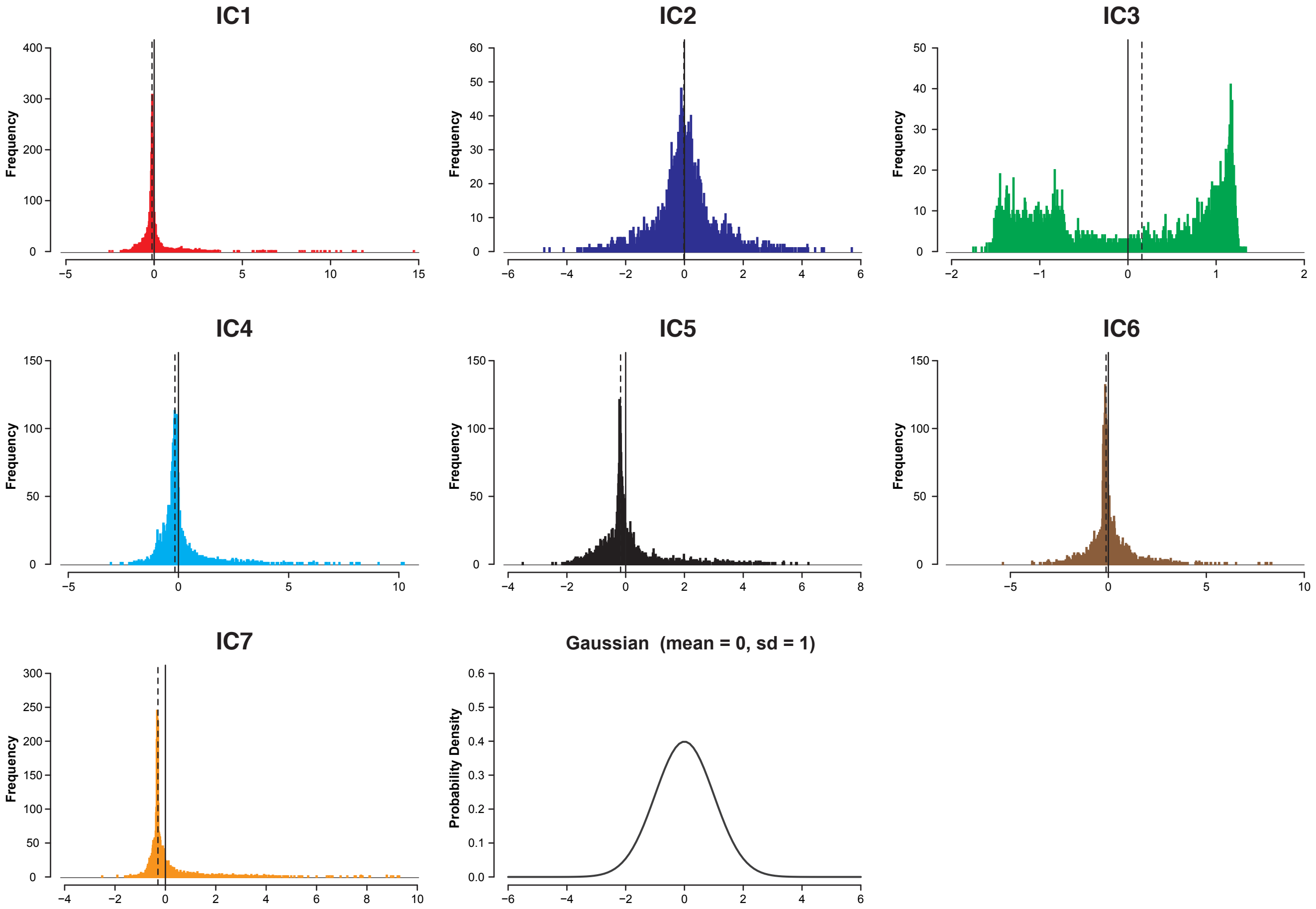

Supplementary Figure S5. Frequency distribution of the seven ICs extracted in this study. The horizontal axis corresponds to each IC score. The vertical solid and dashed lines represent mean and median values for each IC, respectively. Because each IC is constrained to have unit variance in the fastICA algorithm, an IC score of 1 corresponds to 1 standard deviation along each IC. A Gaussian distribution of zero mean and unit variance is also shown for comparison.

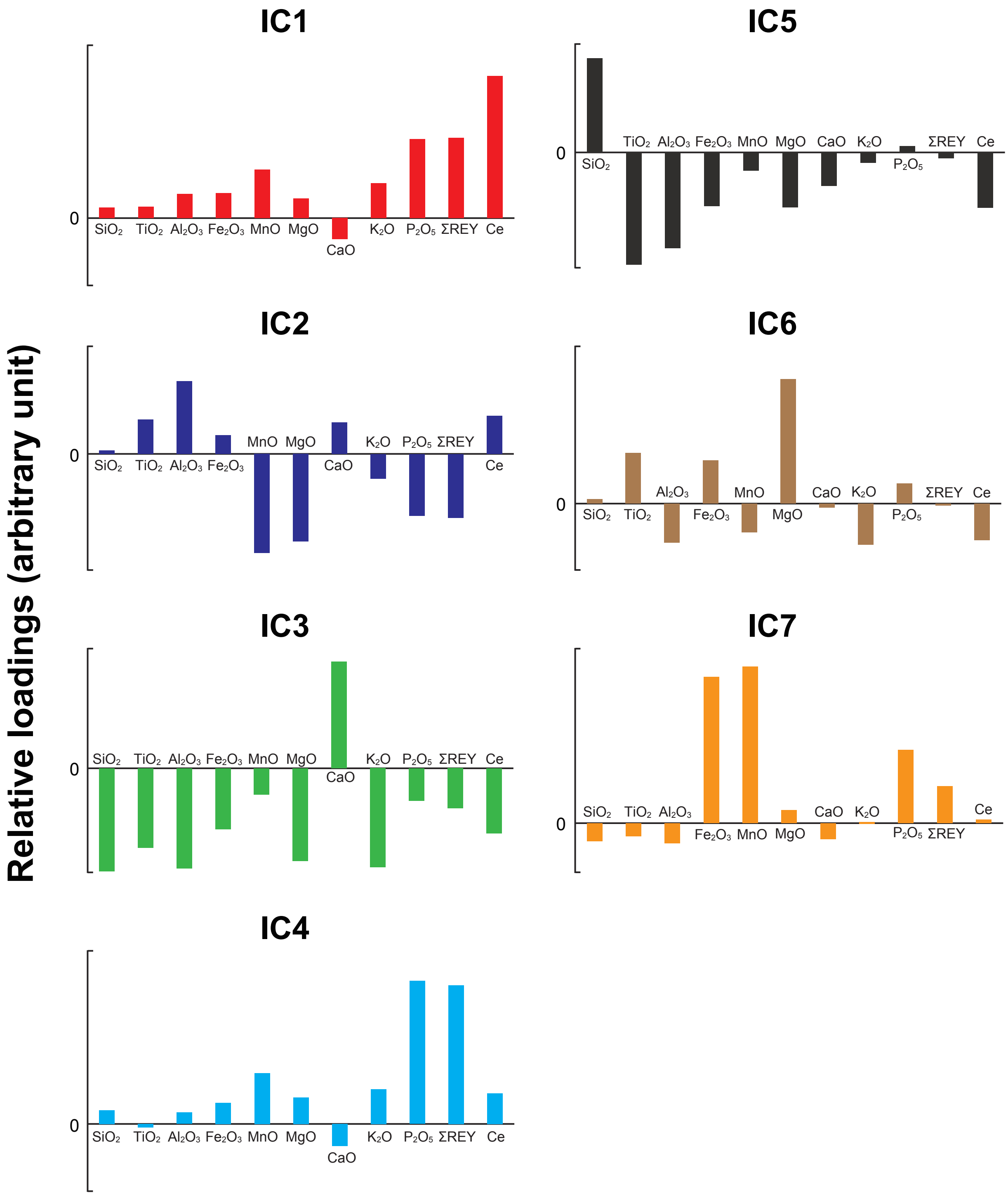

Supplementary Figure S6. Relative loadings of each element for IC1 to IC7. The relative loading of the j-th element for the i-th IC can be obtained by dividing a_{ij}, or the (i, j) component of the mixing matrix $\mathbf{A}$, by a sample standard deviation of the j-th element.

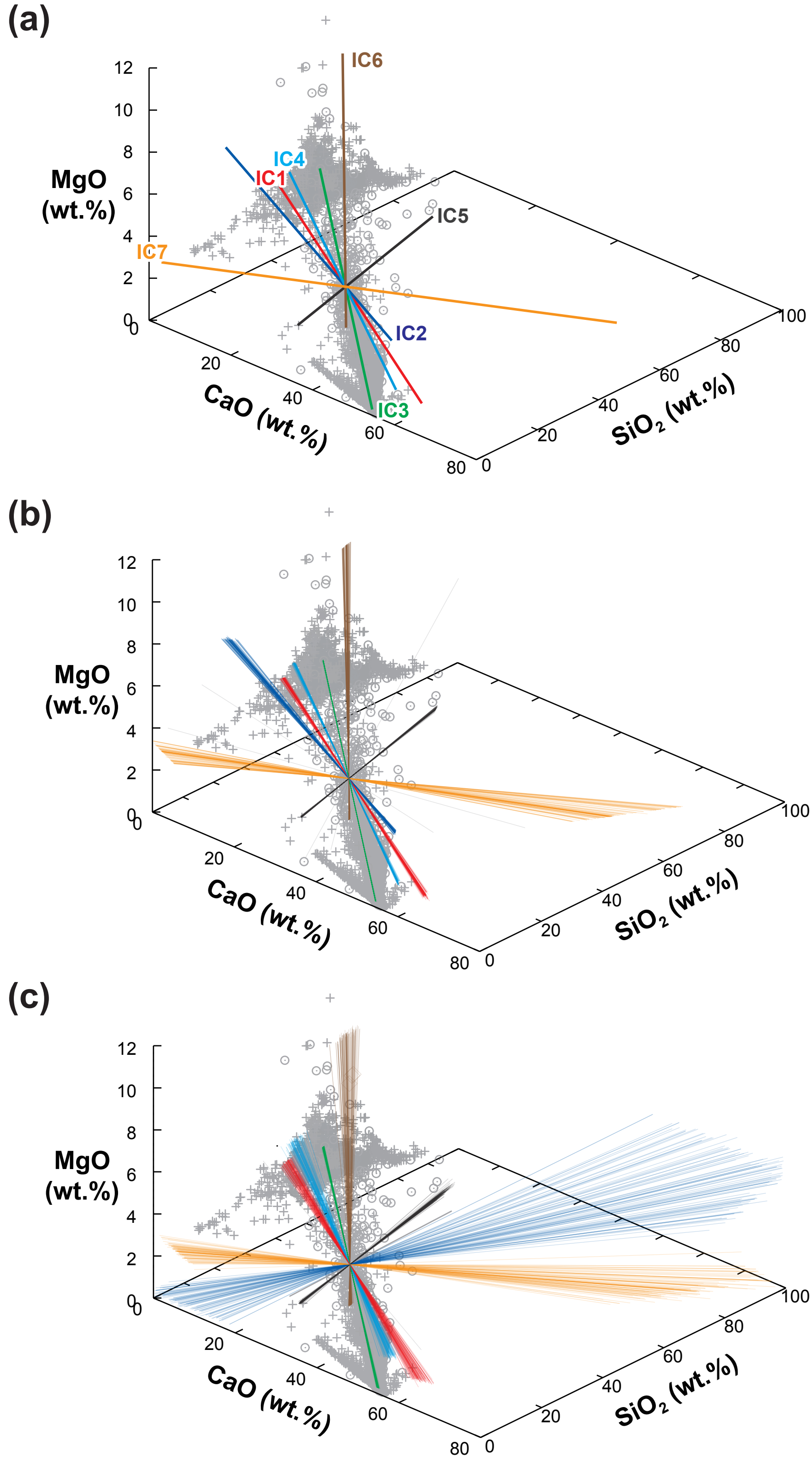

Supplementary Figure S7. Three-dimensional views of the compositional space of SiO_2–CaO–MgO. All of the sediment data are shown in gray. (**a**) IC vectors discussed in the text from a different angle as that shown in Fig. 2a. (**b**) Results of 100 computational runs for evaluating the effects of random initial values given in the fastICA algorithm to estimate the matrix **W**. All ICs showed well-converged slopes, indicating that the ICs have robustness to built-in random valuables of the algorithm. (**c**) Results of other 100 runs using synthetic perturbed datasets to evaluate the effects of data uncertainties such as analytical error. The slopes of ICs still converge well except for IC2 (dark blue), which shows slopes that different from b and much less convergence than other ICs. This suggests that IC2 is not robust to data uncertainty.

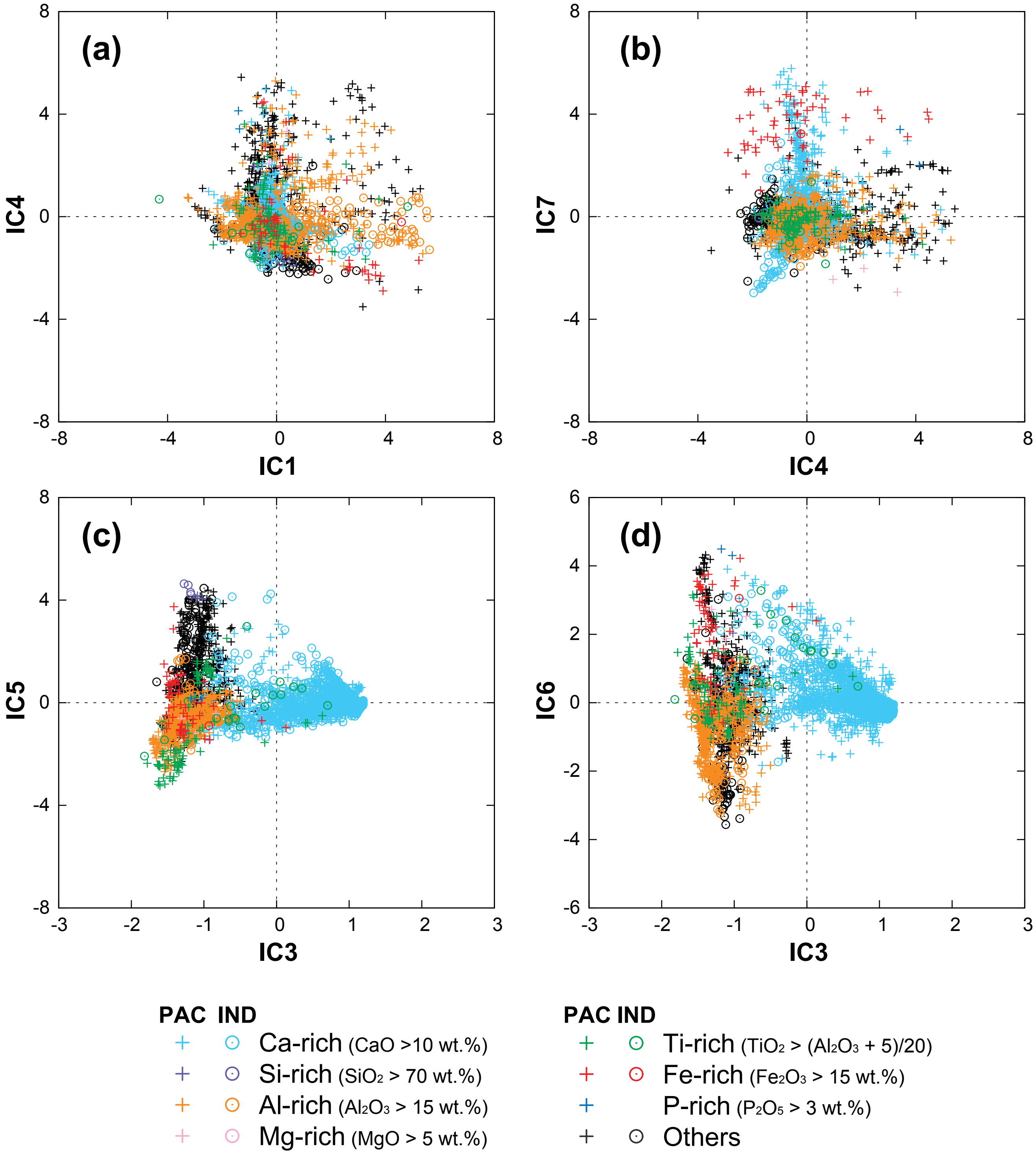

Supplementary Figure S8. Data distribution in selected 2-dimensional IC spaces. **(a)** IC1 vs. IC4, **(b)** IC4 vs. IC7, **(c)** IC3 vs. IC5, and **(d)** IC3 vs. IC6. Plotted are the result of ICA that excluded samples with high-IC-scores (>3) in the result discussed in the main text. IC3 ranges from −1.7 to 1.3, and does not show a long-tailed distribution like the other ICs (e.g. IC1; see Supplementary Figure S5). Therefore, no outlier is considered for IC3.

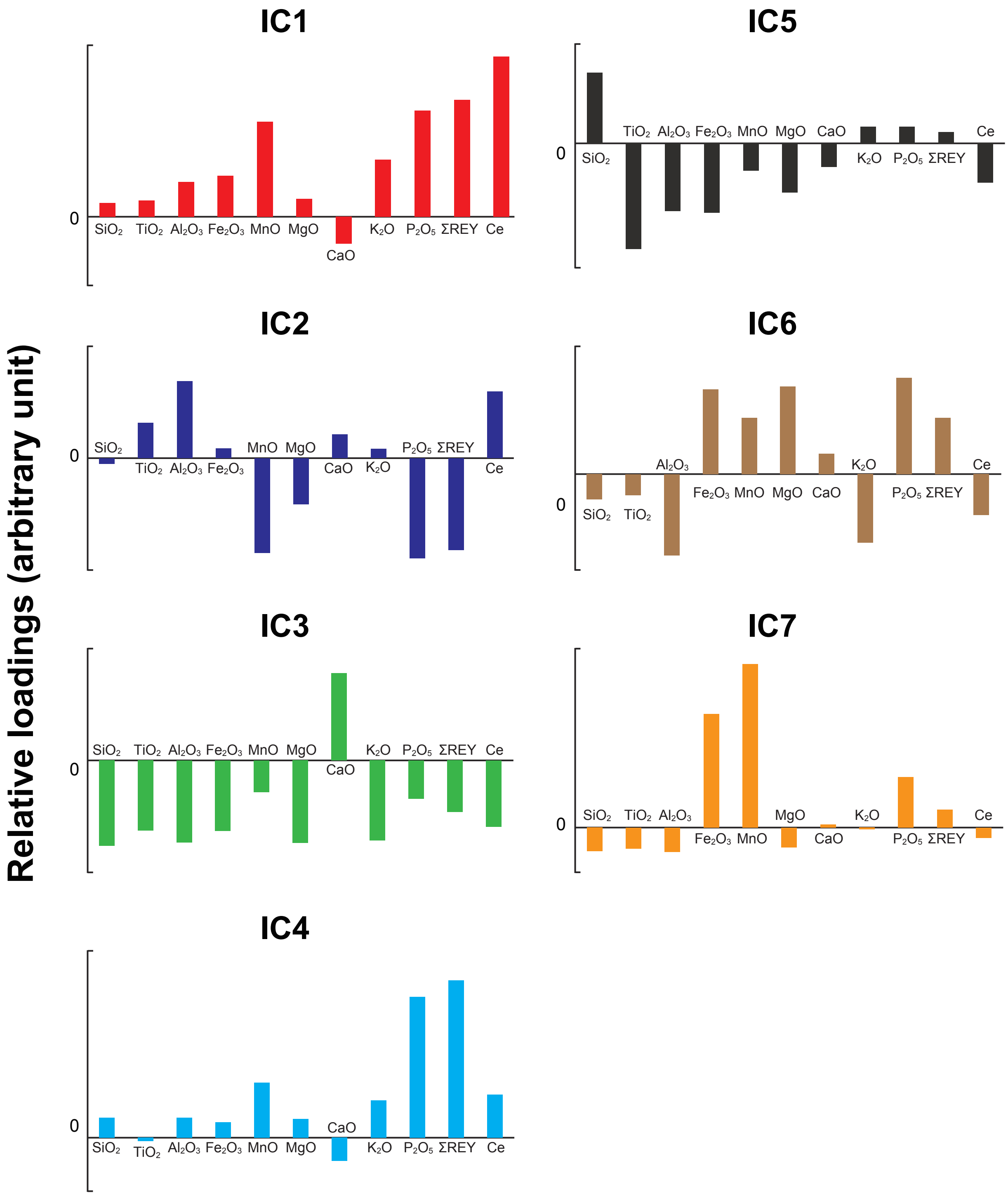

Supplementary Figure S9. Relative loadings of each element for IC1 to IC7 in the result of ICA excluding samples having high-IC-scores (>3 except for IC3) in the result discussed in the main text.

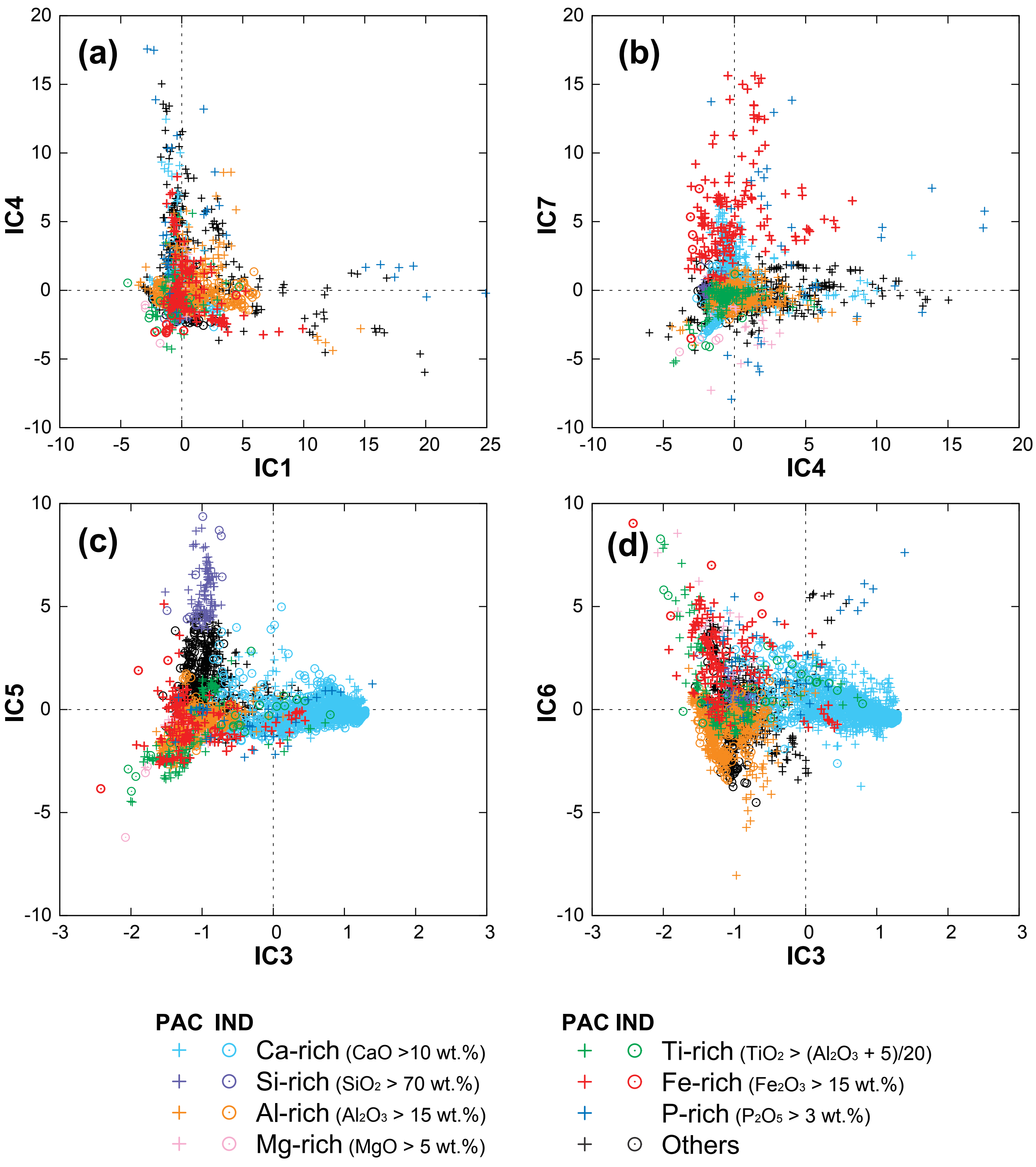

Supplementary Figure S10. Data distribution in selected 2-dimensional IC spaces. **(a)** IC1 vs. IC4, **(b)** IC4 vs. IC7, **(c)** IC3 vs. IC5, and **(d)** IC3 vs. IC6. Plotted are the projection of all data into the IC space using the whitening matrix and the un-mixing matrix estimated from the data set excluding high-IC-samples.

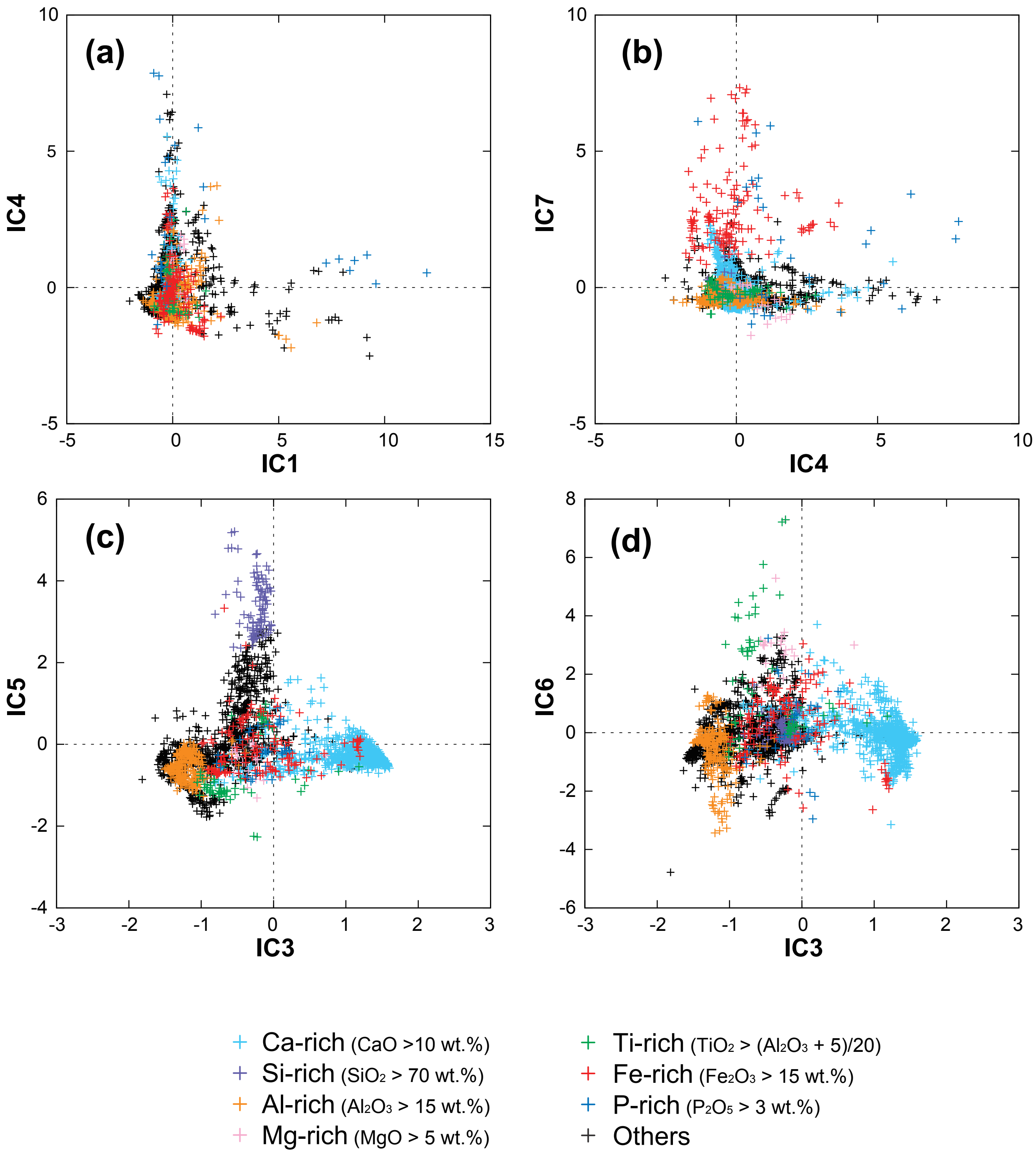

Supplementary Figure S11. Data distribution in selected 2-dimensional IC spaces. **(a)** IC1 vs. IC4, **(b)** IC4 vs. IC7, **(c)** IC3 vs. IC5, and **(d)** IC3 vs. IC6. Plotted are the result of ICA using the Pacific Ocean samples only.

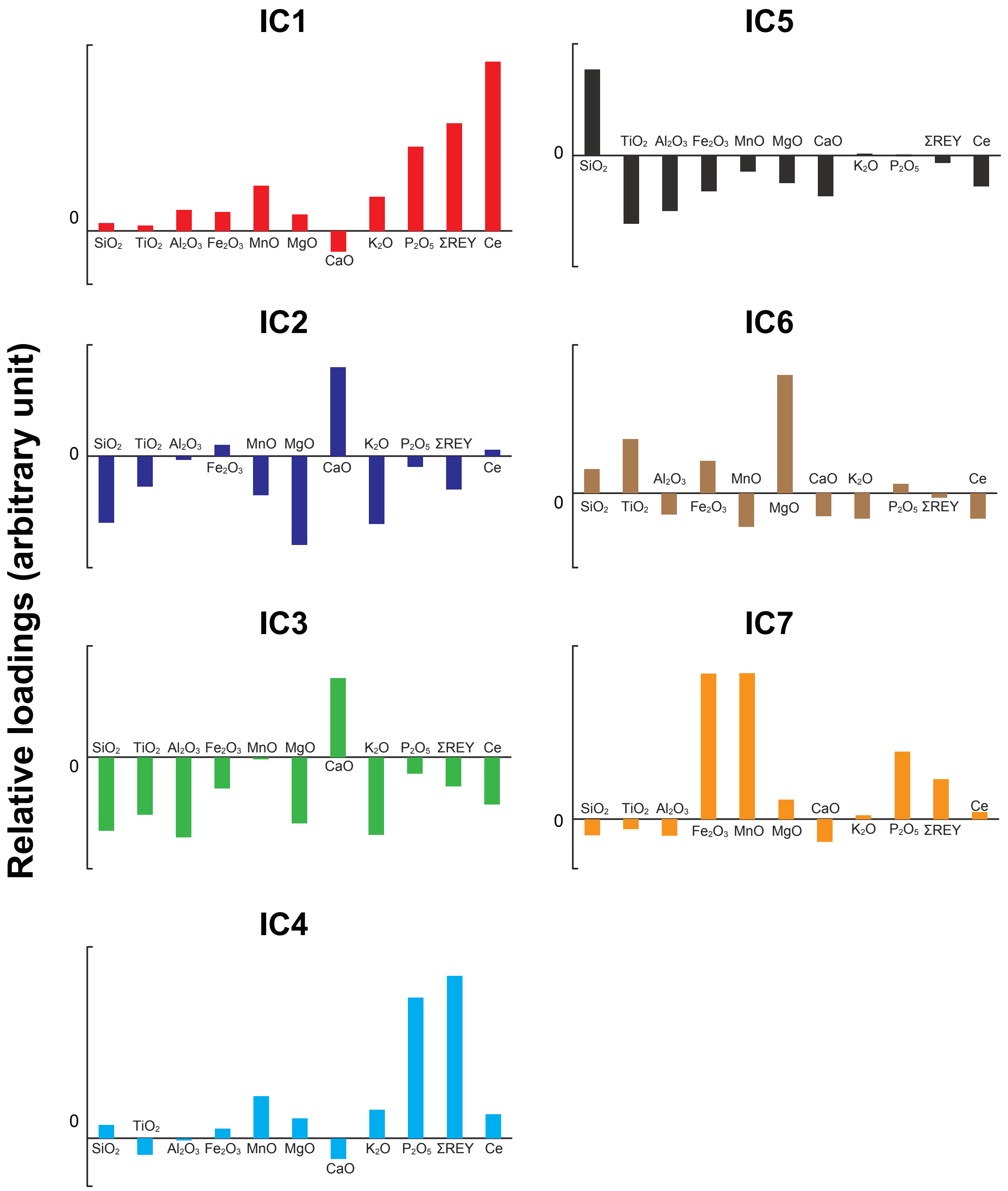

Supplementary Figure S12. Relative loadings of each element for IC1 to IC7 in the result of ICA using the Pacific Ocean samples only.

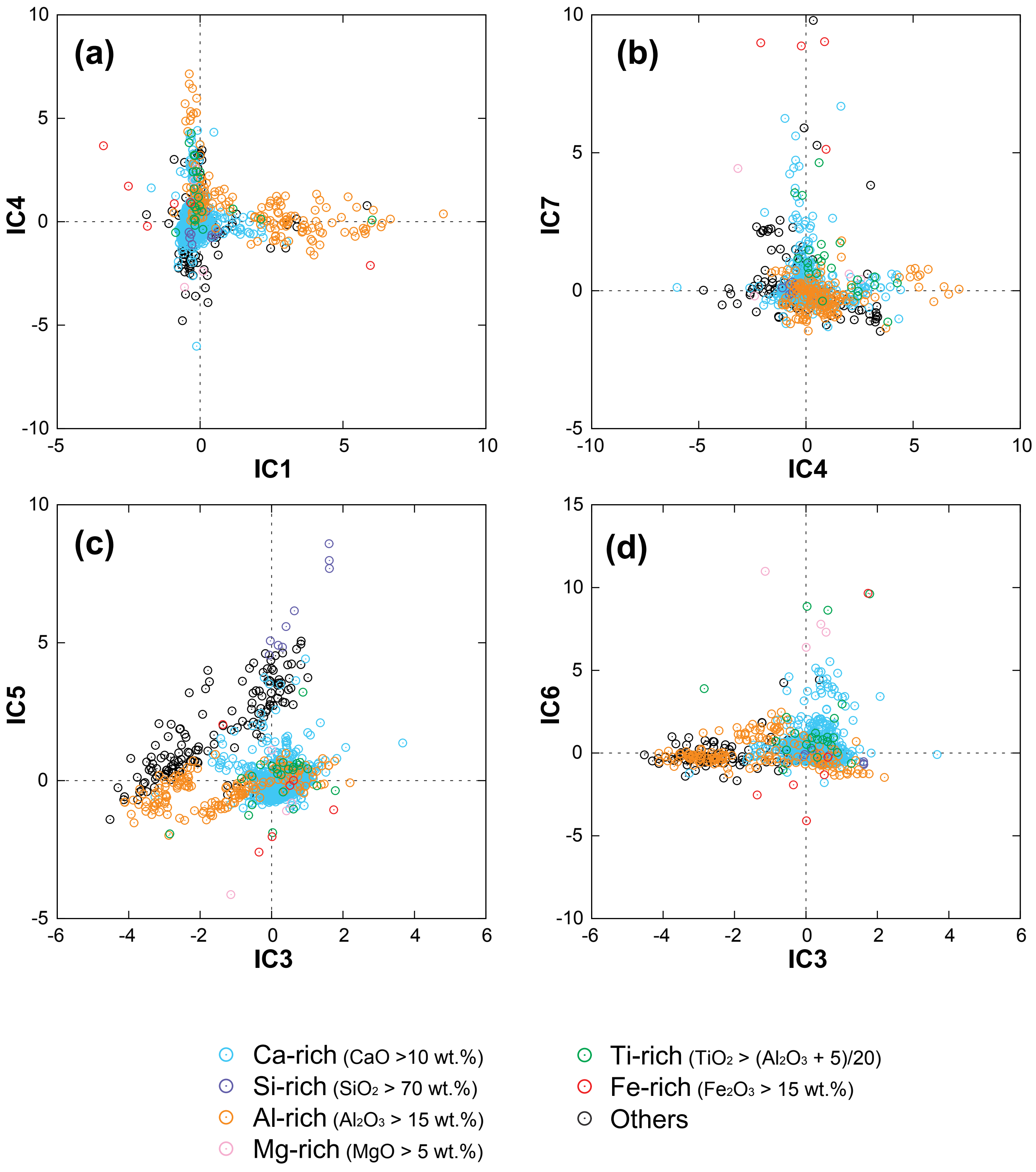

Supplementary Figure S13. Data distribution in selected 2-dimensional IC spaces. **(a)** IC1 vs. IC4, **(b)** IC4 vs. IC7, **(c)** IC3 vs. IC5, and **(d)** IC3 vs. IC6. Plotted are the result of ICA using the Indian Ocean samples only.

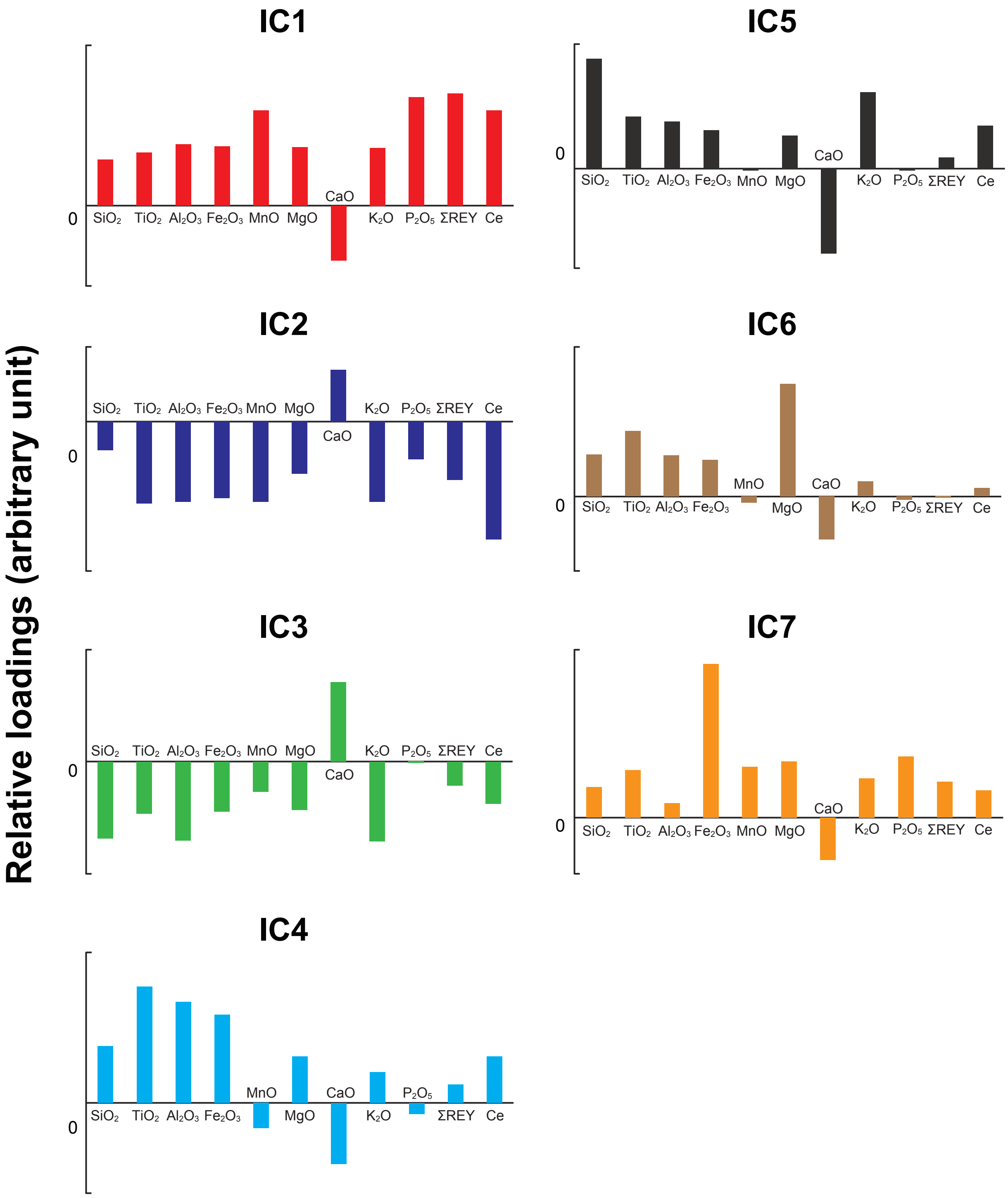

Supplementary Figure S14. Relative loadings of each element for IC1 to IC7 in the result of ICA using the Indian Ocean samples only.

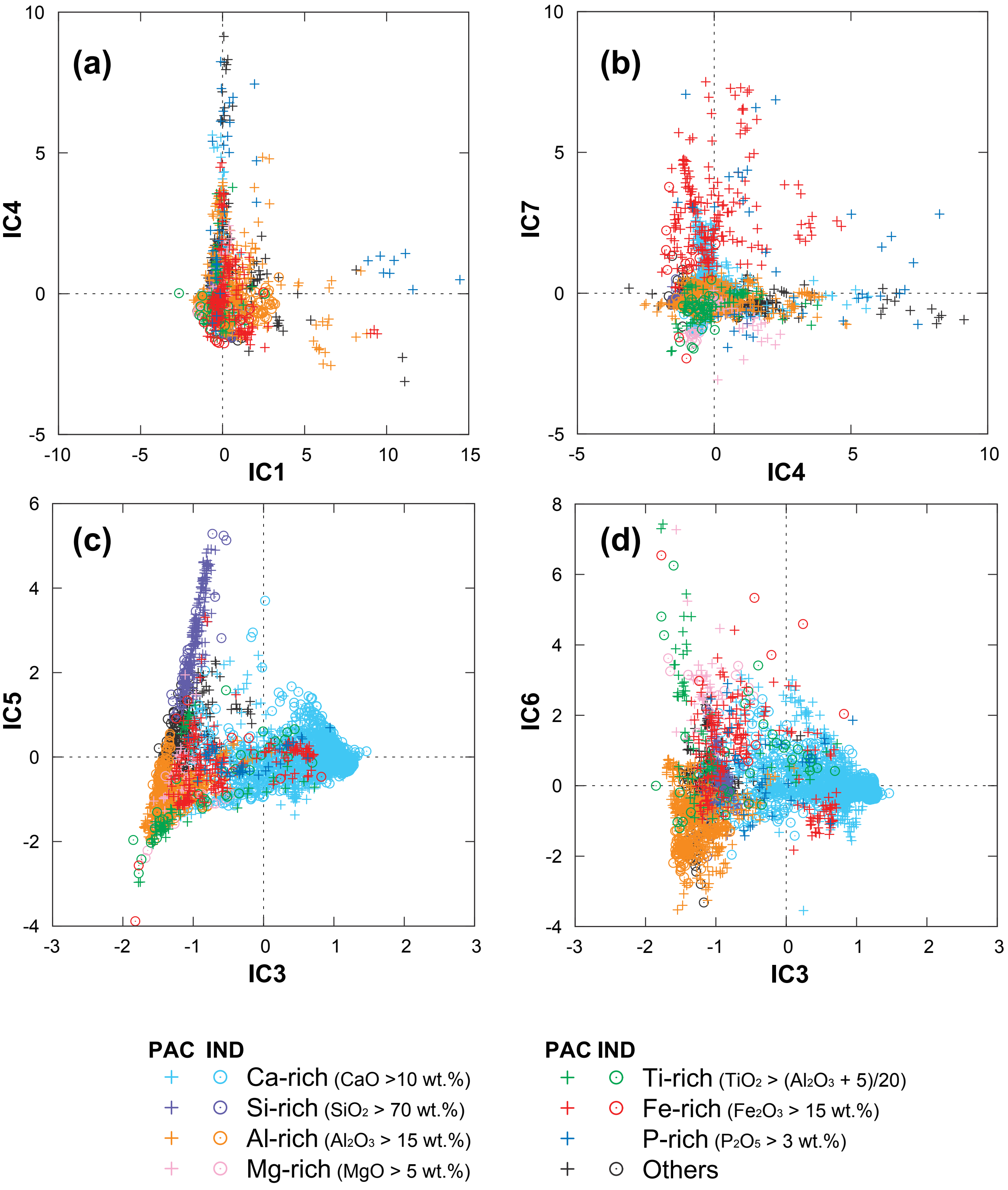

Supplementary Figure S15. Data distribution in selected 2-dimensional IC spaces. **(a)** IC1 vs. IC4, **(b)** IC4 vs. IC7, **(c)** IC3 vs. IC5, and **(d)** IC3 vs. IC6. Plotted are the result of ICA using XRF raw data of nine major elements before being normalized to 100%, including Na_2O content and loss on ignition.

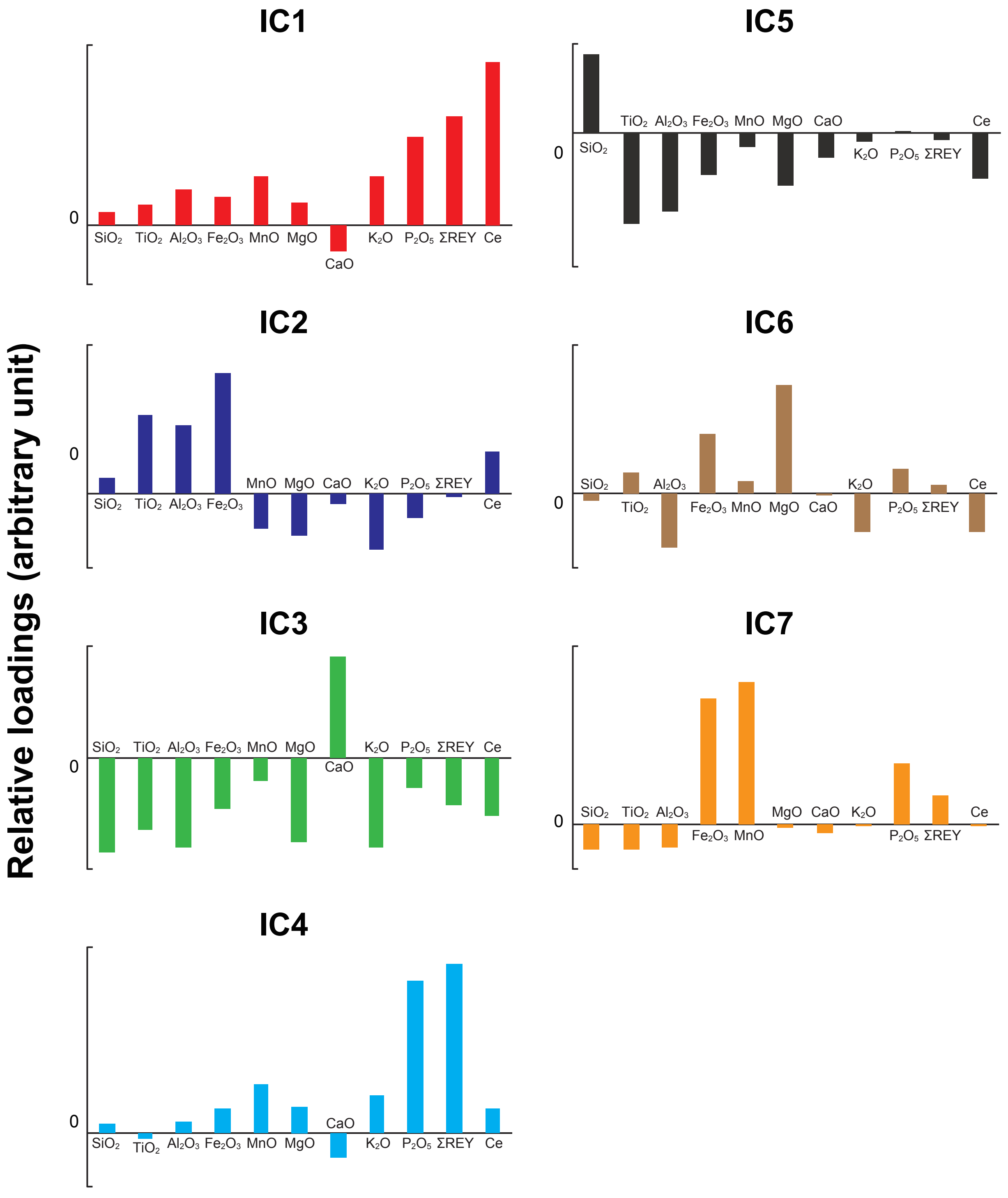

Supplementary Figure S16. Relative loadings of each element for IC1 to IC7 in the result of ICA using XRF raw data of nine major elements before being normalized to 100% including Na_2O content and loss on ignition.

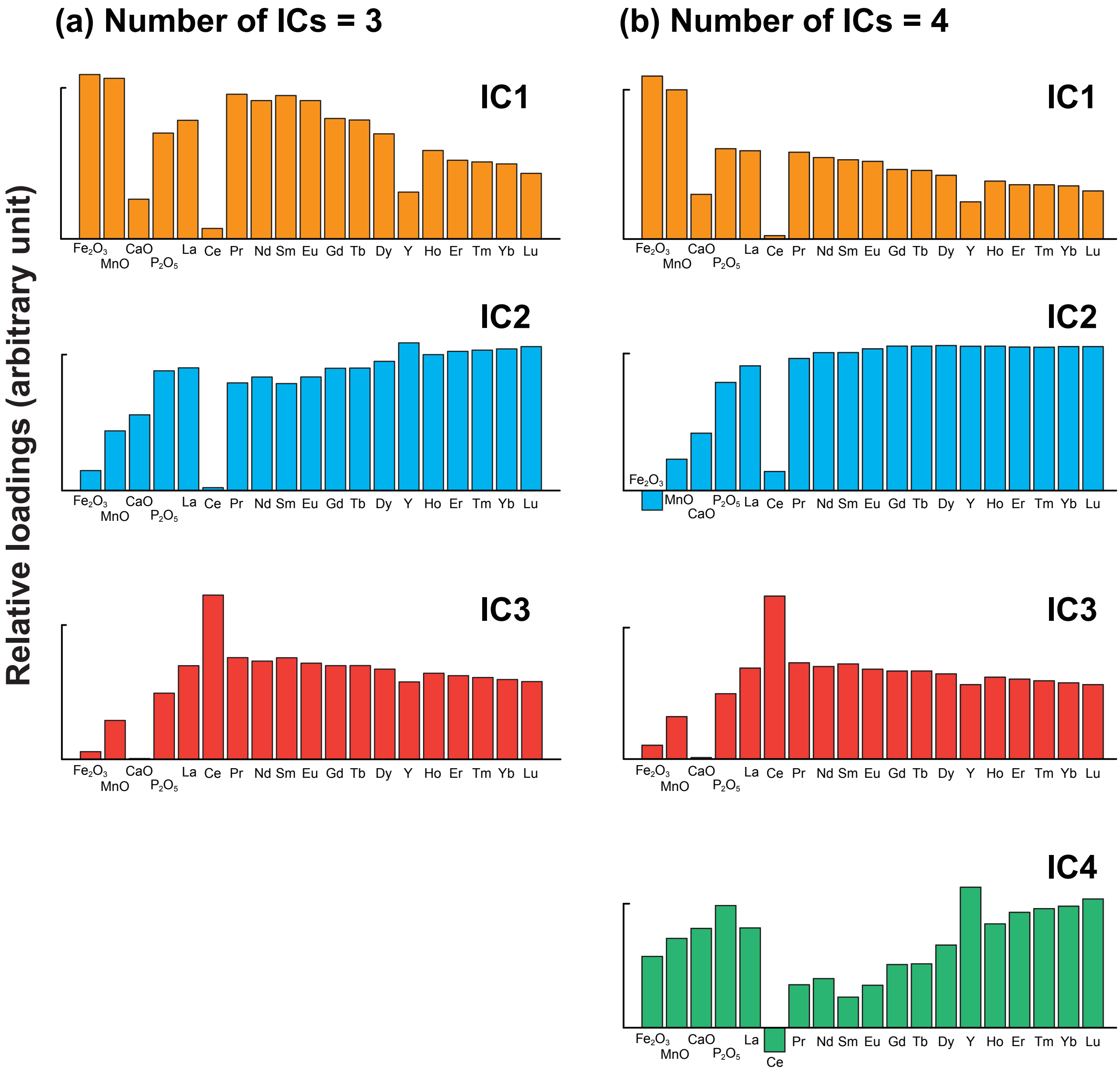

Supplementary Figure S17. Relative loadings of each element for the ICs in the result of ICA using a sample subset, excluding high-Ca (CaO > 10wt.%) and high-Si samples (SiO_2 > 70 wt.%), with the selected elements Fe_2O_3, MnO, CaO, P_2O_5, and all of the separated REY elements. (**a**) and (**b**) are the results in the case where the number of extracted ICs are 3 and 4, respectively.

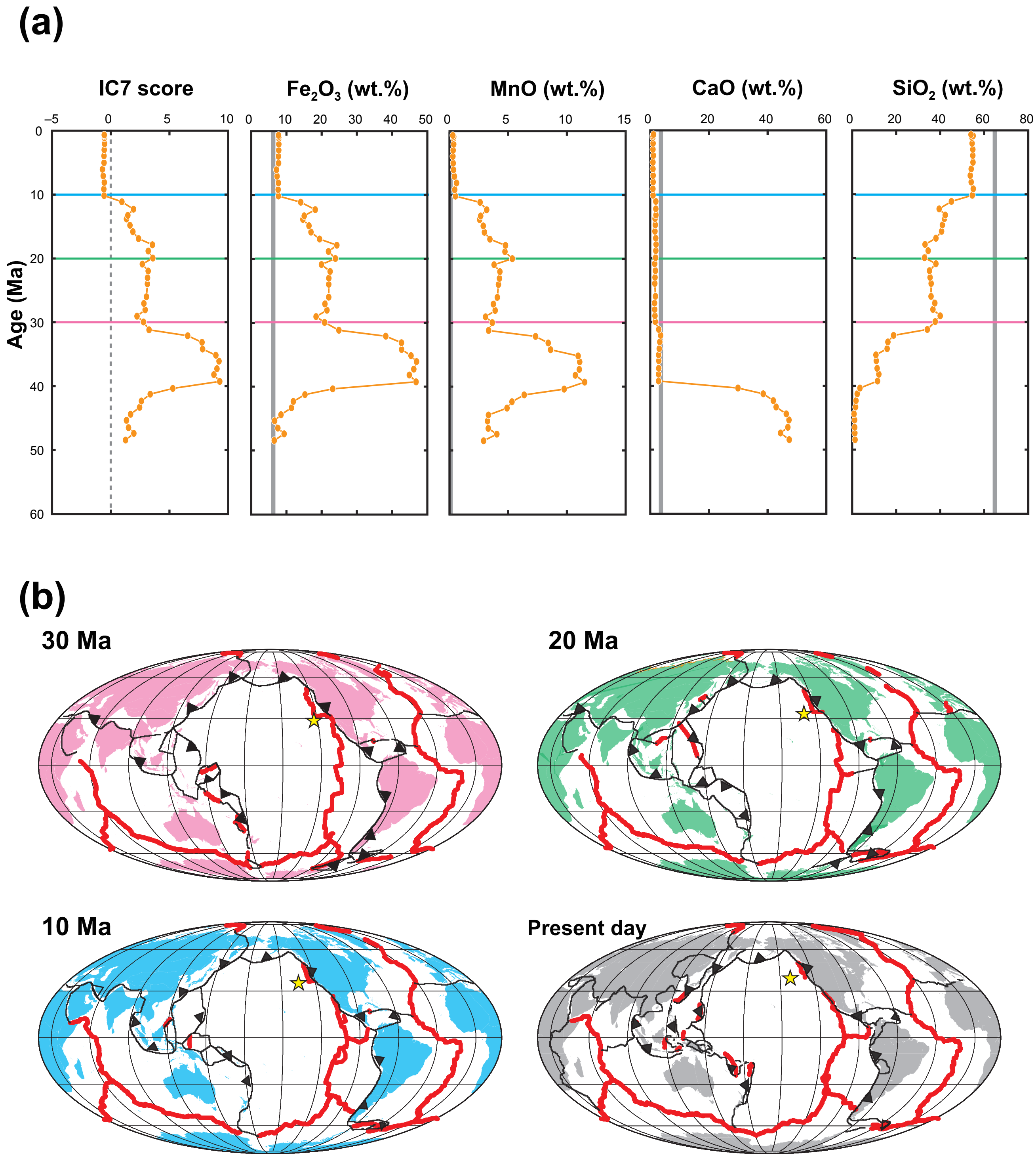

Supplementary Figure S18. **(a)** Downhole variations of IC7 scores and major element contents at DSDP Site 38 in the eastern North Pacific Ocean. Vertical gray solid lines indicate the values of the North American Shale Composite (Ref. S16). Horizontal colored lines correspond to the time slices shown in (**b**). Age model is based on Ref. S10. **(b)** Paleogeographic reconstructions of Mid Ocean Ridges (red solid lines) and approximate paleopositions of DSDP Site 38 (yellow stars) since 30 Ma. The past distibutions of ridges are from Ref. S27. These maps were created using the Generic Mapping Tools software (Ref. S28; https://www.soest.hawaii.edu/gmt/), Version 4.5.8 and the GPlates software (Ref. S13 and S14; http://www.gplates.org), Version 1.2.0.

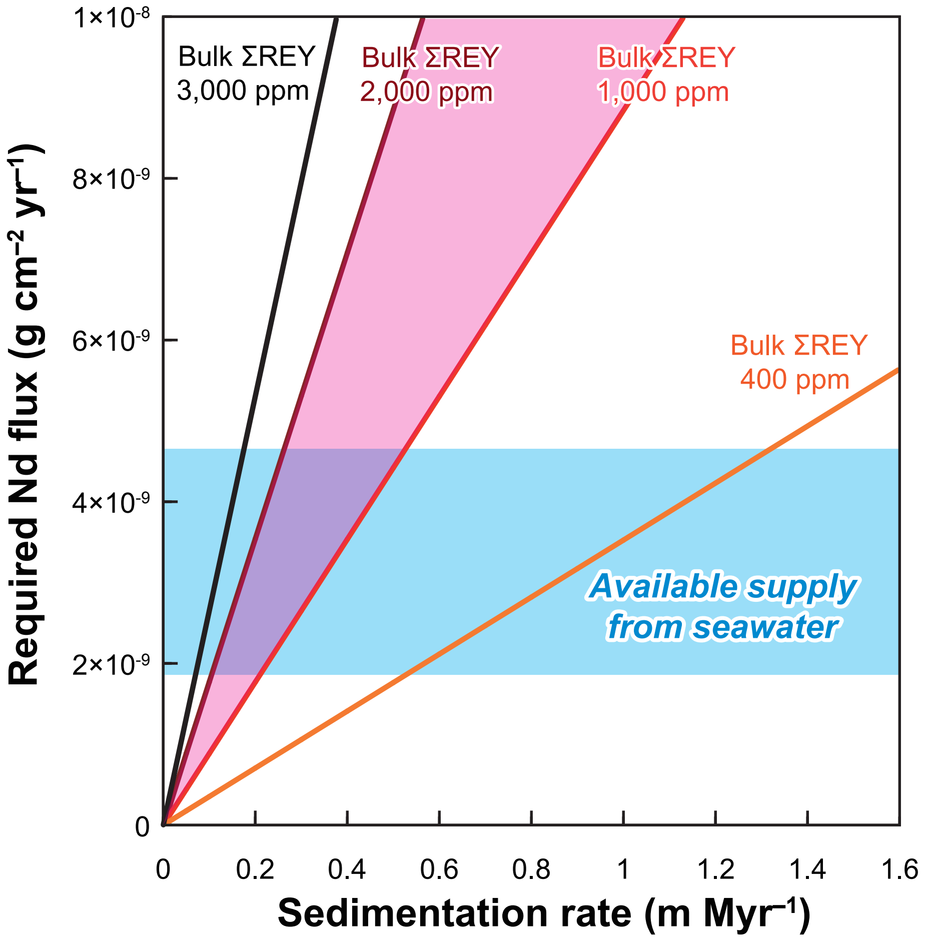

Supplementary Figure S19. Flux of Nd required to form REY-rich mud as a function of the sedimentation rate. The assumed water depth is 5,000 m, which is the typical depth for the occurrence of REY-rich mud. The slopes of the lines changed with the bulk ΣREY content. The horizontal blue bars indicate available Nd supplies from the overlying seawater. The pink areas represent REY-rich mud with relatively high resource potential (bulk ΣREY = 1,000–2,000 ppm).

Supplementary Table S1. Estimated loadings for each IC and standard deviations of each elemental content for original data. The loadings correspond to a mixing matrix **A**.

	SiO_2	TiO_2	Al_2O_3	Fe_2O_3	MnO	MgO	CaO	K_2O	P_2O_5	ΣREY	Ce
IC1	1.37	0.02	0.80	0.73	0.36	0.15	-2.56	0.28	0.23	134	44.5
IC2	0.13	0.02	0.67	0.15	-0.21	-0.19	1.07	-0.05	-0.05	-29.7	3.34
IC3	-22.7	-0.29	-5.63	-3.06	-0.33	-1.21	22.1	-1.35	-0.16	-114.4	-34.7
IC4	1.43	-0.01	0.30	0.50	0.31	0.16	-2.18	0.22	0.34	188	7.75
IC5	7.46	-0.15	-1.93	-0.97	-0.08	-0.26	-2.50	-0.05	0.01	-6.08	-10.68
IC6	0.36	0.07	-0.79	0.78	-0.13	0.58	-0.31	-0.20	0.04	-0.62	-7.03
IC7	-2.30	-0.03	-0.66	4.30	1.16	0.10	-1.92	0.01	0.22	61.6	1.05
Standard deviation of each elemental content (n = 3,968)	24.1	0.39	6.14	5.50	1.39	1.42	22.6	1.49	0.55	312	58.5

Supplementary Table S2. Relative loadings for each IC. Each value is obtained by deviding original loadings in Table S1 by a standard deviation of each element.

	SiO_2	TiO_2	Al_2O_3	Fe_2O_3	MnO	MgO	CaO	K_2O	P_2O_5	ΣREY	Ce
IC1	0.057	0.060	0.130	0.133	0.260	0.104	-0.113	0.186	0.422	0.430	0.760
IC2	0.006	0.051	0.109	0.028	-0.148	-0.131	0.047	-0.037	-0.092	-0.095	0.057
IC3	-0.942	-0.727	-0.917	-0.557	-0.241	-0.850	0.978	-0.907	-0.297	-0.367	-0.594
IC4	0.059	-0.015	0.049	0.092	0.221	0.115	-0.096	0.150	0.621	0.601	0.133
IC5	0.310	-0.369	-0.315	-0.177	-0.059	-0.181	-0.110	-0.034	0.021	-0.020	-0.183
IC6	0.015	0.167	-0.129	0.142	-0.094	0.409	-0.014	-0.135	0.066	-0.002	-0.120
IC7	-0.095	-0.069	-0.107	0.782	0.837	0.069	-0.085	0.007	0.392	0.198	0.018

Supplementary Table S3. Result of Principal Component Analysis of bulk sediment composition data from Pacific and Indian Oceans.

	Eigenvalue	Proportion of variance	Cumulative proportion
PC1	6.861	0.6238	0.6238
PC2	1.937	0.1761	0.7999
PC3	0.788	0.0717	0.8715
PC4	0.526	0.0479	0.9194
PC5	0.361	0.0328	0.9522
PC6	0.172	0.0156	0.9678
PC7	0.123	0.0112	0.9789
PC8	0.089	0.0081	0.9871
PC9	0.074	0.0067	0.9938
PC10	0.066	0.0060	0.9998
PC11	0.003	0.0002	1.0000

Supplementary Table S4. Compiled sedimentation rates, ΣREY contents and dry bulk densities for REY-rich layers at representative sites having high-IC1 and high-IC4 values.

Site	Type	Sedimentation rate (m/Myr)	Bulk ΣREY* (ppm, including Ce)	Dry bulk density (g/cm^3)	Reference
DSDP Site 213	IC1	N/A	900–1,100	0.47–0.71	von der Borch et al. (1974)
DSDP Site 596	IC1	0.19–0.27	1,000–2,800	0.53–0.59	Zhou and Kyte (1992); Lyle (2003)
ODP Site 801	IC1 & IC4	0.58	1,000–2,000	0.42	Lyle (2003)
DSDP Site 68	IC4	0.1	~1,100	0.86	Lyle (2003)
DSDP Site 74	IC4	0–0.98	1,300–2,000	0.32–0.44	Lyle (2003)
DSDP Site 597	IC4	0.12	~1,600	0.55	Lyle (2003)
ODP Site 800	IC4	0.52	~1,600	0.48	Lyle (2003)

* This study
